# Role of Polyunsaturated Fat in Modifying Cardiovascular Risk Associated With Family History of Cardiovascular Disease: Pooled De Novo Results From 15 Observational Studies

**DOI:** 10.1161/CIRCULATIONAHA.123.065530

**Published:** 2023-12-04

**Authors:** Federica Laguzzi, Agneta Åkesson, Matti Marklund, Frank Qian, Bruna Gigante, Traci M. Bartz, Julie K. Bassett, Anna Birukov, Hannia Campos, Yoichiro Hirakawa, Fumiaki Imamura, Susanne Jäger, Maria Lankinen, Rachel A. Murphy, Mackenzie Senn, Toshiko Tanaka, Nathan Tintle, Jyrki K. Virtanen, Kazumasa Yamagishi, Matthew Allison, Ingeborg A. Brouwer, Ulf De Faire, Gudny. Eiriksdottir, Luigi Ferrucci, Nita G. Forouhi, Johanna M. Geleijnse, Allison M Hodge, Hitomi Kimura, Markku Laakso, Ulf Risérus, Anniek C. van Westing, Stefania Bandinelli, Ana Baylin, Graham G. Giles, Vilmundur Gudnason, Hiroyasu Iso, Rozenn N. Lemaitre, Toshiharu Ninomiya, Wendy S. Post, Bruce M. Psaty, Jukka T. Salonen, Matthias B. Schulze, Michael Y. Tsai, Matti Uusitupa, Nicholas J. Wareham, Seung-Won Oh, Alexis C. Wood, William S. Harris, David Siscovick, Dariush Mozaffarian, Karin Leander

**Affiliations:** Unit of Cardiovascular and Nutritional Epidemiology, Institute of Environmental Medicine (F.L., A.A., U.D.F., K.L.), Karolinska Institutet, Stockholm, Sweden.; Cardiovascular Medicine Unit, Department of Medicine Solna (B.G.), Karolinska Institutet, Stockholm, Sweden.; Department of Epidemiology, Johns Hopkins Bloomberg School of Public Health, Baltimore, MD (M.M., W.S.P).; The George Institute for Global Health, Faculty of Medicine, University of New South Wales, Sydney, Australia (M.M.).; Department of Public Health and Caring Sciences, Clinical Nutrition and Metabolism, Uppsala University, Sweden (M.M., U.R).; Section of Cardiovascular Medicine, Boston Medical Center and Boston University Chobanian and Avedisian School of Medicine, MA (F.Q.).; Department of Nutrition (F.Q.), Boston, MA.; Harvard T.H. Chan School of Public Health (H.C.), Boston, MA.; Cardiovascular Health Research Unit, Departments of Biostatistics (T.M.B.), University of Washington, Seattle.; Medicine (T.M.B., R.N.L., B.M.P.), University of Washington, Seattle.; Epidemiology (B.M.P.), University of Washington, Seattle.; Health Systems and Population Health (B.M.P.), University of Washington, Seattle.; Cancer Epidemiology Division, Cancer Council Victoria, Melbourne, Australia (J.K.B., A.M.H., G.G.G.).; Department of Molecular Epidemiology, German Institute of Human Nutrition Potsdam-Rehbruecke, Nuthetal (A.K.B., S.J., M.B.S.).; German Center for Diabetes Research, Neuherberg (A.K.B., S.J., M.B.S.).; Departments of Epidemiology and Public Health and Medicine and Clinical Science, Graduate School of Medical Sciences, Kyushu University, Fukuoka, Japan (Y.H., T.N.).; Medical Research Council Epidemiology Unit, University of Cambridge School of Clinical Medicine, UK (F.I., N.G.F., N.J.W.).; Institutes of Public Health and Clinical Nutrition (M. Lankinen, J.K.V., M.U.), University of Eastern Finland, Kuopio.; Clinical Medicine, Internal Medicine (M. Laakso), University of Eastern Finland, Kuopio.; Kuopio University Hospital (M. Laakso), University of Eastern Finland, Kuopio.; Cancer Control Research, BC Cancer Agency, Vancouver, Canada (R.A.M.).; School of Population and Public Health, University of British Columbia, Vancouver, Canada (R.A.M.).; United States Department of Agriculture/Agricultural Research Service Children’s Nutrition Research Center, Baylor College of Medicine, Houston, TX (M.S., A.C.W.).; Longitudinal Study Section, National Institute on Aging, Baltimore, MD (T.T., L.F.).; Fatty Acid Research Institute, Sioux Falls, SD (N.T., W.S.H.).; Department of Population Health Nursing Science, University of Illinois – Chicago (N.T.).; Department of Public Health Medicine, Institute of Medicine (K.Y., H.K.), University of Tsukuba, Japan.; Health Services Research and Development Center (K.Y., H.K.), University of Tsukuba, Japan.; Department of Family Medicine, University of California, San Diego, La Jolla (M.A.).; Department of Health Sciences, Faculty of Science, Vrije Universiteit Amsterdam, The Netherlands (I.A.B.).; Amsterdam Public Health Research Institute, The Netherlands (I.A.B.).; Icelandic Heart Association, Kopavogur (G.E., V.G.).; Division of Human Nutrition and Health, Wageningen University and Research, The Netherlands (J.M.G., A.C.v.W.).; Centre for Epidemiology and Biostatistics, University of Melbourne, Victoria, Australia (A.M.H., G.G.G.).; Geriatric Unit, Azienda Unità Sanitaria Locale Toscana Centro, Florence, Italy (S.B.).; University of Michigan School of Public Health, Ann Arbor (A. Baylin).; Precision Medicine, School of Clinical Sciences at Monash Health, Monash University, Victoria, Australia (G.G.G.).; Faculty of Medicine, University of Iceland, Reykjavik (V.G.).; Public Health, Department of Social Medicine, Osaka University Graduate School of Medicine, Suita, Japan (H.I.).; Institute for Global Health Policy Research, Bureau of International Health Cooperation, National Center for Global Health and Medicine, Tokyo, Japan (H.I.).; Department of Medicine, Division of Cardiology, Johns Hopkins University School of Medicine, Baltimore, MD (W.S.P.).; Metabolic Analytical Services Oy, Helsinki, Finland (J.T.S.).; University of Helsinki, the Faculty of Medicine, Department of Public Health, Finland (J.T.S.).; Institute of Nutritional Science, University of Potsdam, Nuthetal, Germany (M.B.S.).; Department of Laboratory Medicine and Pathology, University of Minnesota, Minneapolis (M.Y.T.).; Department of Family Medicine, Seoul National University College of Medicine, and Healthcare System Gangnam Center, Seoul National University Hospital, Republic of Korea (S.W.O.).; Department of Internal Medicine, Sanford School of Medicine, University of South Dakota, Sioux Falls (W.S.H.).; The New York Academy of Medicine, New York (D.S.).; Food Is Medicine Institute, Friedman School of Nutrition Science and Policy, Tufts University, Boston, MA (D.M.).

**Keywords:** biomarkers, cardiovascular diseases, family medical history, polyunsaturated fatty acids, precision medicine

## Abstract

**BACKGROUND:**

It is unknown whether dietary intake of polyunsaturated fatty acids (PUFA) modifies the cardiovascular disease (CVD) risk associated with a family history of CVD. We assessed interactions between biomarkers of low PUFA intake and a family history in relation to long-term CVD risk in a large consortium.

**METHODS:**

Blood and tissue PUFA data from 40 885 CVD-free adults were assessed. PUFA levels ≤25th percentile were considered to reflect low intake of linoleic, alpha-linolenic, and eicosapentaenoic/docosahexaenoic acids (EPA/DHA). Family history was defined as having ≥1 first-degree relative who experienced a CVD event. Relative risks with 95% CI of CVD were estimated using Cox regression and meta-analyzed. Interactions were assessed by analyzing product terms and calculating relative excess risk due to interaction.

**RESULTS:**

After multivariable adjustments, a significant interaction between low EPA/DHA and family history was observed (product term pooled RR, 1.09 [95% CI, 1.02–1.16]; *P*=0.01). The pooled relative risk of CVD associated with the combined exposure to low EPA/DHA, and family history was 1.41 (95% CI, 1.30–1.54), whereas it was 1.25 (95% CI, 1.16–1.33) for family history alone and 1.06 (95% CI, 0.98–1.14) for EPA/DHA alone, compared with those with neither exposure. The relative excess risk due to interaction results indicated no interactions.

**CONCLUSIONS:**

A significant interaction between biomarkers of low EPA/DHA intake, but not the other PUFA, and a family history was observed. This novel finding might suggest a need to emphasize the benefit of consuming oily fish for individuals with a family history of CVD.

**Clinical Perspective**

**What Is New?**

- This study investigated whether the cardiovascular disease (CVD) risk associated with a family history of CVD is modified by a diet low in n-3 or n-6 polyunsaturated fatty acids, a research question that has not been well established.
- Based on a harmonized pooled analysis of de novo results from 15 observational studies involving 40 885 individuals across 10 different countries, using blood or tissue measurements of polyunsaturated fatty acids as surrogate markers of dietary intake, a statistically significant interaction between low eicosapentaenoic/docosahexaenoic acids, but not linoleic acid and alpha linolenic acid, and a family history of CVD was observed.

**What Are the Clinical Implications?**

- Low blood or tissue levels of n-3 polyunsaturated fatty acids, reflecting a low intake of oily fish, were observed to enhance the CVD risk associated with a family history of CVD.
- This study suggests that individuals with a family history of CVD may benefit even more from recommendations to consume food rich in eicosapentaenoic/docosahexaenoic acids.
- Although a family history of CVD is a nonmodifiable CVD risk factor, there appears to be potential to limit its adverse effects.

Despite preventive efforts and therapeutic interventions, cardiovascular disease (CVD) is still the leading cause of morbidity and mortality in most countries.^1^ Organizations such as the American Heart Association have emphasized the importance of primary prevention in combatting the burden of CVD across the world.^2^ An increased identification of susceptible groups for targeted preventive measures is an important step towards a more proactive system of prevention.^3–7^ Further, with tailored interventions, improved adherence to preventive measures can be obtained.^8,9^

The current guidelines for CVD prevention acknowledge family history of CVD as a nonmodifiable risk factor that calls for heightened attention to modifiable risk factors such as smoking, hypertension and hyperlipidemia.^3,10^ Whether special emphases should also be placed on dietary interventions (beyond those to address the latter risk factors) for this high-risk group is not directly addressed in current guidelines,^3,10,11^ and there is limited knowledge on whether family history of CVD calls for targeted dietary advice.^12^

Among the dietary factors, particular attention in CVD prevention has been given to the quantity and type of fat consumed.^13^ High intake of n-3 and n-6 polyunsaturated fatty acids (PUFA), from oily fish and vegetable oils or nuts, has been consistently recommended for the prevention of CVD, while a balance between energy intake and expenditure is maintained.^10,11^ Beneficial associations of the n-3 PUFA eicosapentaenoic acid (EPA) and docosahexaenoic acid (DHA) with CVD have been observed in epidemiological studies,^14–16^ and EPA/DHA have lowered risk in some, but not all, clinical trials.^17^ More inconsistent findings have been reported for the essential, plant-based PUFA (ie, n-6, linoleic acid [LA]^15,18–20^ and n-3, alpha-linolenic acid [ALA]),^18,21–24^ although recent evidence seems to also indicate a protective role for these fatty acids in relation to CVD.^15,19,21,24^ With few exceptions, intake of PUFA worldwide is generally lower^25–27^ than the 5% to 11% of total energy intake commonly recommended.^28^

CVD tends to aggregate in families, a phenomenon partly explained by a genetic component of CVD, as demonstrated in twin studies^29,30^ and partly appears to be explained by an aggregation of traditional CVD risk factors.^31,32^ Individuals with ≥1 family member with CVD are at higher risk of CVD compared with those without a family history of CVD, and the risk is even higher if the event occurred at a younger age.^33,34^ The reported prevalence of family history of CVD varies largely over different studies (2% to 30%)^33–35^ depending on how it is defined (ie, which first degree relative [parent, sibling, or child] is affected, as well as the ages and number of the affected first-degree relatives.^33,34^ To our knowledge, no study to date has examined biomarkers of PUFA in combination with data on family history to assess interactions.

In this study, we aimed to assess whether the risk of CVD in individuals with a family history of CVD would be increased by a diet low in PUFA to a greater extent than in those without such a history. We used blood and tissue PUFA biomarkers as surrogates for PUFA intakes. We performed harmonized pooled analyses of de novo results from 15 studies in the Fatty Acids and Outcomes Research Consortium (FORCE).

## METHODS

### Study Population: FORCE Consortium

FORCE (http://force.nutrition.tufts.edu/) is a scientific collaborative effort aiming to investigate the relationship between fatty acids and several chronic diseases. Details about how this scientific collaboration is practically carried out are described in the Supplemental Material. For the present investigation, all the observational studies which were members of the consortium (N=41) by 2019, regardless of study design, were invited to participate. Inclusion criteria for participation were availability of biomarkers of PUFA intake (LA, ALA, EPA, or DHA), data on family history of CVD, and data on CVD diagnoses and causes of death. In total, 15 studies (11 cohort studies, 1 case–cohort study, 2 nested case–control study and 1 case–control study) across 10 countries (Australia, Costa Rica, Finland, Germany, Iceland, Italy, Japan, Sweden, the United Kingdom, and the United States) were included. A uniform analysis protocol was formed and distributed to each participating study. Participants >18 years of age or those with a previous diagnosis of coronary heart disease (CHD) or ischemic stroke were excluded from the analyses. All participating studies had institutional ethical approval and informed consent from the study participants.

### Family History of CVD

Family history of CVD was defined as having a first-degree relative (parent or sibling) affected by fatal or non-fatal CVD (CHD or stroke), irrespective of the relative’s age at diagnosis (definition A). In sensitivity analyses, we used a family history definition that accounts for the relative’s age at diagnosis (definition B). For both definitions, the reference category consisted of individuals without any first degree relative affected by CVD. Thus, for the analyses using definition B, individuals meeting definition A but not B were excluded from the analyses. All 15 participating studies were included in the analyses that used the family history A definition. Thirteen participating studies collected information about CVD in both parents and siblings. Two studies specified that only full siblings were considered; the remaining studies did not address full- versus half-siblings. One study had collected information about CVD in siblings only. One study asked about the presence of family history of CVD without further specification on which first-degree relative was affected. For the definition of a family history of CVD, most of the studies considered both myocardial infarction and stroke events in the first-degree relative, whereas 2 studies considered only myocardial infarction and one study considered only CHD. In addition to myocardial infarction and stroke, one study also considered hypertension. Seven participating studies were included in the analyses that used the family history B definition. The age cut-off for CVD in the first-degree relative varied across the participating cohorts and was not always differentiated by sex (Tables S1 and S2).

### Biomarkers of PUFA Intake

The biomarkers of PUFA intake (n-6 PUFA: LA; n-3 PUFA: ALA, EPA, and DHA) were measured in different lipid compartments (including phospholipids [n=6], red blood cells [n=3], total serum [n=3], plasma [n=1], cholesterol esters [n=1], and adipose tissue [n=1]), as percentages of total fatty acids. Information on the method used to measure fatty acid biomarkers in each of the participating studies is reported in Table S1. Each participating study created 3 different binary variables to reflect low PUFA intake, using the study-specific 25th percentile as cut-off value (≤25th percentile): (1) low LA, (2) low ALA, and (3) low EPA/DHA. A schematic overview of how these variables were created can be found in Table S3. In sensitivity analyses, the study-specific ≤50th percentile was employed as cut-off value to define each of the low PUFA variables.

### Outcome Definition

Incident CVD was defined as a composite of fatal or nonfatal CHD (*International Classification of Diseases, Tenth Revision* [*ICD-10*] codes: I20–I25, I46) and ischemic stroke (*ICD-10* codes I63–I65). Details on CVD assessment are provided in Table S1.

### Covariates

The covariates included in the harmonized analysis protocol were age, sex, geographical location, race, education level, occupation, physical activity, smoking, alcohol intake, prevalent diabetes, prevalent hypertension, prevalent dyslipidemia, body mass index, aspirin use, cod liver/fish oil supplements, biomarker levels of ALA, EPA, and DHA for the analyses of LA and biomarkers of LA and arachidonic acid for the analyses of ALA and EPA/DHA. When the classification of these covariates could not be fully harmonized in accordance with the protocol in a participating cohort, study-specific categories were used (Table S4 through S8). The selection of covariates for use in the harmonized study protocol’s analytical models to adjust for possible confounding was guided by subject knowledge and what was used in previous studies of PUFA in relation to CVD risk (eg, Del Gobbo et al^14^ and Marklund et al^19^), as well as in previous research on interactions between PUFA and family history.^12^ The choice was made after balancing what was considered practical and possible (Supplemental Material). For missing covariates, a missing indicator category was used for categorical covariates; for missing continuous covariates, each was handled either by imputation or exclusion as decided by each study investigator (Table S1).

### Statistical Analysis

For prospective cohort studies, multivariable-adjusted Cox proportional hazards models, with robust variance, were used to estimate the hazard ratios of CVD, while for case–cohort designs we used weighted Cox regression models. Follow-up time was calculated from baseline (when sampling took place) to the date of the CVD event, end of follow-up, lost to follow-up, or death, whichever occurred first. For nested case–control studies and case–control studies with risk-set sampling, conditional or unconditional multivariable-adjusted logistic regression was employed, as appropriate, to estimate odds ratios as proxies of relative risk (RR).

Interactions were evaluated by assessing departure from additivity^36–38^ (on an additive scale), occurring when the combined effect of 2 exposures is larger (or smaller) than the sum of the individual effects and departure from multiplicativity (on a multiplicative scale), occurring when the combined effect of 2 exposures is larger (or smaller) than the product of the individual effects.^39^ Hazard ratios or odds ratios of CVD, both interpreted as RR and recorded as beta coefficients, were estimated in each participating study for 3 dummy variables: (1) double exposed: low PUFA with family history of CVD (ie, dummy 1); (2) single exposed: low PUFA without family history of CVD (ie, dummy 2); and (3) single exposed: family history of CVD without low PUFA (ie, dummy 3). The reference category was always the group with neither of the 2 exposures. In addition, in each participating study a regression analysis encompassing the product term “low PUFA × family history” was performed. The 3 dummy variables and the product term were created for each of the 2 definitions of family history of CVD (definitions A and B) and for each of the low PUFA categories (ie, EPA/DHA, LA, and ALA). All models were adjusted for the covariates included in the harmonized analysis protocol as previously described.

#### Pooled Analysis

Inverse-variance weighted (fixed-effect) meta-analysis^40,41^ was employed to pool each of the 3 study specific hazard ratios or odds ratios of CVD, here referred to as RR, constituent dummy variables as described. These pooled estimates formed the basis for the analysis of interaction. For interaction on an additive scale, relative excess risk due to interaction (RERI)^36,38^ was calculated as follows:


$$\begin{array}{l} {\text{Pooled RR}}_{\text{double exposed}}-\\ {\text{pooled RR}}_{\text{single exposed to low PUFA}}-\\ {\text{pooled RR}}_{\text{single exposed to family history}}+\;1. \end{array}$$

A 95% CI for the RERI was computed using the delta method elaborated on by Hosmer and Lemeshow,^42^ in which the elements of the covariance matrix of the estimate coefficients from each of the participating studies are meta-analyzed and used to calculate the standard errors. For assessment of interaction on a multiplicative scale, the RR for the product term low PUFA × family history, recorded as beta coefficients at the study level, were pooled using inverse-variance weighted (fixed-effect) meta-analysis.

Heterogeneity was assessed using the Cochran Q test,^40^ where *P* values <0.05 were considered statistically significant, and the I^2^ statistic.^43^

To assess the robustness of our results, we performed leave-one-out meta-analysis. Sensitivity analyses were also performed by lipid compartments in which PUFA were measured.

To give an idea of the possible meaning of the results of the study from a public health perspective, when relevant, we calculated proportion of cases that could be attributable to the single and double exposures. The formula used accounts for the strengths of the associations observed and the proportion of CVD cases that are exposed (p_c_): [(pooled RR−1) / pooled RR)] × p_c_.

Pooled analyses were performed using SAS 9.4 (SAS Institute) and STATA 12.1 (Stata Corp) statistical software.

## RESULTS

Pooled analyses included a total of 40 885 individuals, among whom 7945 first-time CVD events occurred during follow-up (applicable to cohort, nested case–control, and case–cohort design studies) or at recruitment (applicable to a case–control study; Table). At baseline, the average age was 62.7 years (range across the cohorts, 49.1–76.5 years). Approximately half of the included participants were women (range across the cohorts, 0–100%) and the median follow-up time was 12.3 years (range across the cohorts, 7.1–23.4 years). Details of the descriptive characteristics for each participating cohort, for the entire sample and stratified for family history of CVD, are presented in the Table.

**Table.**
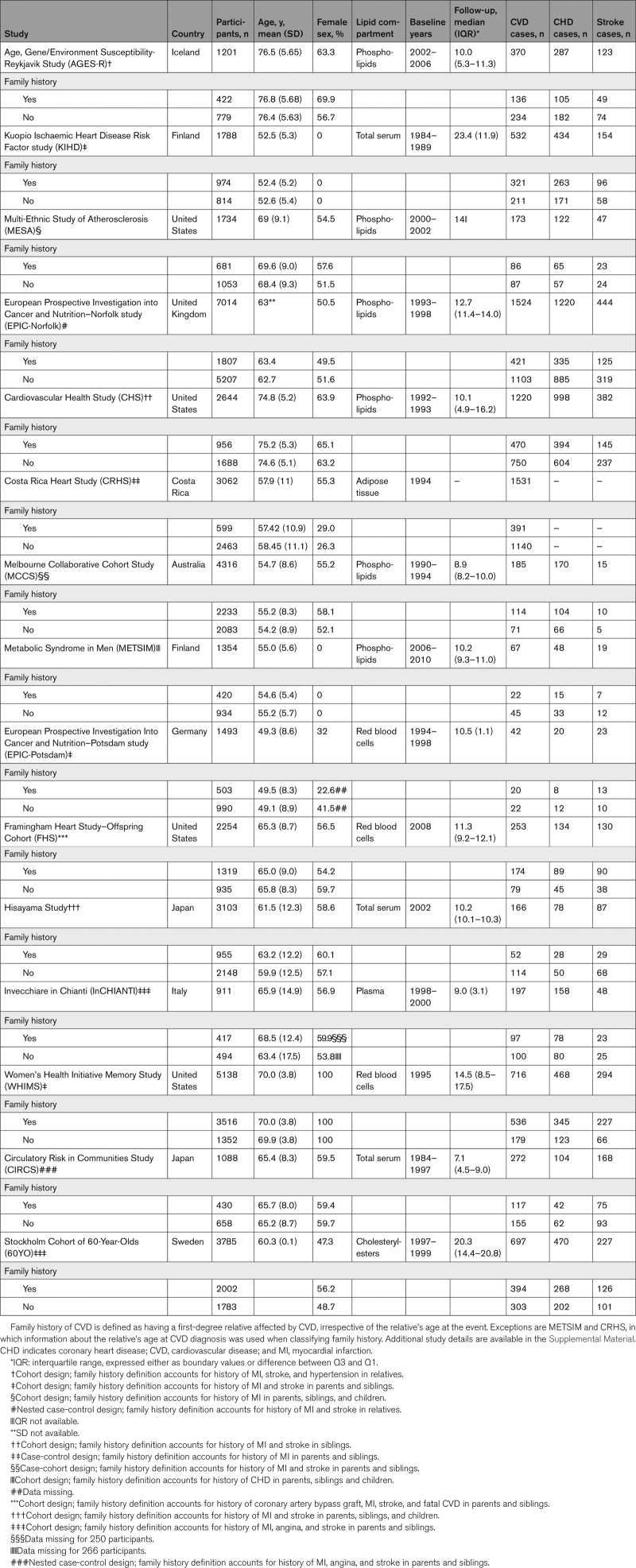
Baseline Characteristics of Participating Study Cohorts by Family History of Cardiovascular Disease

Of the included participants, 15 888 (39%) had a family history of CVD (ie, definition A, based on a definition that does not take into account the relative’s age at diagnosis). Of these, 6126 (14.9%) had a family history of CVD (ie, definition B, based on a definition that does account for the relative’s age at diagnosis).

Distributions of study-specific circulating and adipose levels of the n-6 and n-3 PUFA by family history of CVD are shown in Tables S4 and S5. The corresponding distributions of covariates are presented in Table S4; S6 through S8.

### Family History Regardless of Relative’s Age at Diagnosis and Low PUFA

**Figure 1.**
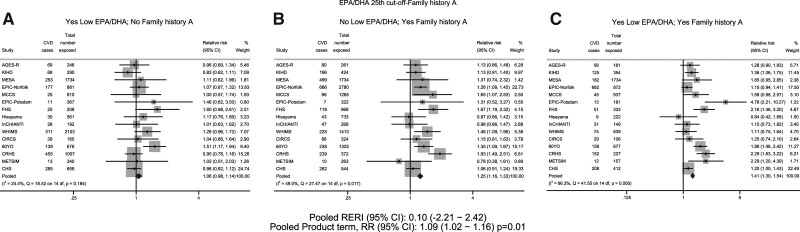
**EPA/DHA cut-off for family history A.** Study-specific and pooled risk estimates for cardiovascular disease in relation to low EPA/DHA (≤25th percentile cut-off) and family history of cardiovascular disease (ie, family history A). **A**, Presence of low EPA/DHA in absence of family history definition A. **B**, Presence of family history A in absence of low EPA/DHA. **C**, Presence of low EPA/DHA, and family history A. **A** through **C**, Reference category consists of individuals with neither low EPA/DHA nor family history A. For pooled analyses, 15 408 individuals formed the reference category; 3408 were cases of cardiovascular disease. 60YO indicates Stockholm Cohort of 60-Year-Olds; AGES-R, Age, Gene/Environment Susceptibility–Reykjavik study; CHS, Cardiovascular Health Study; CIRCS, Circulatory Risk in Communities Study; CRHS, Costa Rica Heart Study; EPIC-Norfolk, European Prospective Investigation into Cancer and Nutrition–Norfolk study; EPIC-Potsdam, European Prospective Investigation into Cancer and Nutrition–Potsdam study; FHS, Framingham Heart Study–Offspring Cohort; Hisayama, Hisayama Study; InCHIANTI, Invecchiare in Chianti; KIHD, Kuopio Ischaemic Heart Disease Risk Factor study; MCCS, Melbourne Collaborative Cohort Study; MESA, Multi-Ethnic Study of Atherosclerosis; METSIM, Metabolic Syndrome in Men; and WHIMS, Women’s Health Initiative Memory Study.

**Figure 2.**
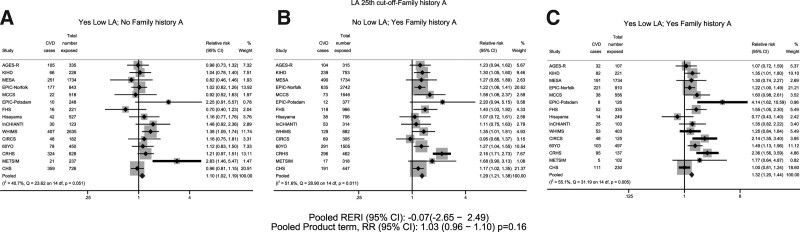
**LA cut-off for family history A.** Study-specific and pooled risk estimates for cardiovascular disease in relation to low LA (≤25th percentile cut-off) and family history of cardiovascular disease (ie, family history A). **A**, Presence of low LA in absence of family history A. **B**, Presence of family history A in absence of low LA. **C**, Presence of low LA and family history A. **A** through **C**, Reference category consists of individuals with neither low LA nor family history A. For pooled analyses, 17 060 individuals formed the reference category; 3665 were cases of cardiovascular disease. 60YO indicates Stockholm Cohort of 60-Year-Olds; AGES-R, Age, Gene/Environment Susceptibility–Reykjavik study; CHS, Cardiovascular Health Study; CIRCS, Circulatory Risk in Communities Study; CRHS, Costa Rica Heart Study; EPIC-Norfolk, European Prospective Investigation into Cancer and Nutrition–Norfolk study; EPIC-Potsdam, European Prospective Investigation into Cancer and Nutrition–Potsdam study; FHS, Framingham Heart Study–Offspring Cohort; Hisayama, Hisayama Study; InCHIANTI, Invecchiare in Chianti; KIHD, Kuopio Ischaemic Heart Disease Risk Factor study; LA, linoleic acid; MCCS, Melbourne Collaborative Cohort Study; MESA, Multi-Ethnic Study of Atherosclerosis; METSIM, Metabolic Syndrome in Men; and WHIMS, Women’s Health Initiative Memory Study.

**Figure 3.**
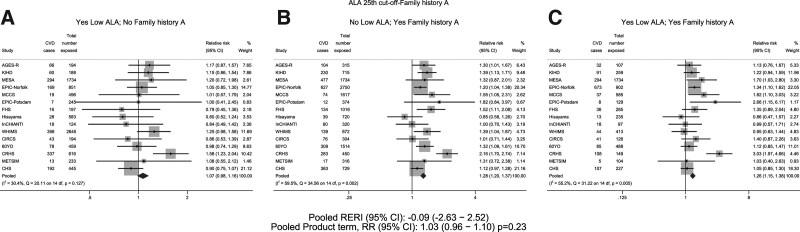
**ALA cut-off for family history A.** Study-specific and pooled risk estimates for cardiovascular disease in relation to low ALA (≤25th percentile cut-off) and family history of cardiovascular disease (ie, family history A). **A**, Presence of low ALA in absence of family history A. **B**, Presence of family history A in absence of low ALA. **C**, Presence of low ALA and family history A. **A** through **C**, Reference category consists of individuals with neither low ALA nor family history A. For pooled analyses, 17 210 individuals formed the reference category; 3715 were cases of cardiovascular disease. 60YO indicates Stockholm Cohort of 60-Year-Olds; AGES-R, Age, Gene/Environment Susceptibility–Reykjavik study; ALA, alpha linolenic acid; CHS, Cardiovascular Health Study; CIRCS, Circulatory Risk in Communities Study; CRHS, Costa Rica Heart Study; EPIC-Norfolk, European Prospective Investigation into Cancer and Nutrition–Norfolk study; EPIC-Potsdam, European Prospective Investigation into Cancer and Nutrition–Potsdam study; FHS, Framingham Heart Study–Offspring Cohort; Hisayama, Hisayama Study; InCHIANTI, Invecchiare in Chianti; KIHD, Kuopio Ischaemic Heart Disease Risk Factor study; MCCS, Melbourne Collaborative Cohort Study; MESA, Multi-Ethnic Study of Atherosclerosis; METSIM, Metabolic Syndrome in Men; and WHIMS, Women’s Health Initiative Memory Study.

Pooled results from the 15 studies included regarding each of the low PUFA variables using the 25th cut-off and a family history of CVD are presented in Figures 1 through 3. Figure 1 shows the pooled results related to low EPA/DHA. Using the group without low EPA/DHA levels and without a family history of CVD as reference category, the analyses of CVD risk yielded the following results: (1) single exposure to low EPA/DHA in absence of a family history (Figure 1A; pooled RR, 1.06 [95% CI, 0.98 – 1.14]); (2) single exposure to a family history in absence of low EPA/DHA (Figure 1B; pooled RR, 1.25 [95% CI, 1.16 – 1.33]); and (3) double exposure to low EPA/DHA in combination with a family history (Figure 1C; pooled RR, 1.41 [95% CI, 1.30–1.54]). Thus, the pooled RR point estimate for the double exposure was greater than the product of, but not the sum of, pooled RR point estimates for the single exposures. The interaction between low EPA/DHA and family history on the multiplicative scale was statistically significant (pooled product term RR, 1.09 [95% CI, 1.02–1.16]; *P*=0.01). However, no significant interaction on the additive scale was observed (pooled RERI RR, 0.10 [95% CI, −2.21 to 2.42]). Figure 2 and Figure 3 show the pooled results related to low LA and ALA, respectively. No significant interaction results were observed either on the multiplicative or the additive scale. The pooled results for the product terms LA × family history and ALA × family history were: RR, 1.03 (95% CI, 0.96–1.10); *P*=0.16 and RR, 1.03 (95% CI, 0.96–1.10); *P*=0.23, respectively, whereas the pooled RERI results were RR, −0.07 (95% CI, −2.65 to 2.49) and RR, −0.09 (95% CI, −2.63 to 2.52).

Results from the sensitivity analysis of interactions using low PUFA cut-off values at the 50th percentile (Figures S1 through S3) were similar to those observed using the 25th percentile; there was evidence of interaction with family history for low EPA/DHA on the multiplicative scale (product term results RR, 1.08 [95% CI, 1.02–1.16]; *P*=0.02), but not for LA nor ALA. No evidence of interactions on the additive scale was found for any of the low PUFA investigated.

### Family History Using Relative’s Age at Diagnosis and Low PUFA

Pooled results from the 7 studies included in the sensitivity analysis, addressing family history definition B in combination with each PUFA, using the 25th cut-off values, are presented in Figures S4 through S6. A borderline statistically significant interaction on the multiplicative scale was observed for EPA/DHA (pooled result of product term EPA/DHA × family history RR, 1.18 [95% CI, 1.00–1.40]; *P*=0.05). No significant interaction on the multiplicative scale was observed for LA or ALA. There were no interactions on the additive scale for any of the PUFA investigated.

Results from sensitivity analyses based on the 50th percentile cut-off (Figures S7 through S9) showed no interactions on either scale.

### Between-Study Heterogeneity

In general, between-study heterogeneity was found to be low-moderate (I^2^ <60%) for the pooled analyses of family history definition A, whereas there was evidence of moderate-high (I^2^ ranged from 50% to 82%) between-study heterogeneity for the pooled analyses of family history definition B.

Results from sensitivity analyses performed with the leave-one-out meta-analysis were similar to those of the corresponding full analysis (data not shown). Results from sensitivity analyses stratified on lipid compartments for PUFA assessment were also in line with the results of the full analysis (data not shown).

### Attributable Proportions

The calculation of attributable proportions showed that the proportion of CVD cases attributed to the double exposure of low EPA/DHA and family history of CVD (definition A) was 5%; for a family history of CVD alone it was 6% and for low EPA/DHA alone it was 1%. The proportion of CVD cases exposed to both a family history of CVD and low EPA/DHA was 18%. For family history alone, it was 28%, and for low EPA/DHA alone, it was 19%.

## DISCUSSION

In this harmonized pooled analysis of de novo results from 15 primarily prospective epidemiological studies, a significant interaction between a family history of CVD and PUFA biomarkers indicating a low intake of oily fish was observed. This result is based on assessments of interaction defined as departure from multiplicativity of effects. At the same time, we observed no significant result when assessing interaction as departure from additivity of effects, although the results point in the same direction. This latter approach contributes quantification of exposure–outcome associations in single-exposed and double-exposed groups, which helps answering the research question and facilitate clinical interpretations.^37^ For the interaction analyses involving low n-3 EPA/DHA, the RR point estimate for the double exposure was clearly higher than the product but not the sum of single exposures; interestingly, this pattern was not observed for the other PUFA biomarkers we studied. For the other PUFA biomarkers, no indications of interactions were observed, regardless of the definition of interaction. Together, these new findings suggest that low PUFA reflecting low consumption of oily fish amplifies the risk associated with having a family history of CVD. Thus, our findings suggest that advice to consume more oily fish should be especially emphasized for individuals with a family history of CVD. Assuming causality behind the observed interaction between low n-3 EPA/DHA and family history of CVD, based on our new results, 5% of the CVD cases could be attributed to the double exposure of low EPA/DHA and family history of CVD.

A side finding from our results that form the basis for the assessment of interactions suggests a link between low intake of n-3 EPA/DHA, ALA and n-6 LA and increased risk of CVD in individuals regardless of their CVD family history. However, in individuals without a family history of CVD, only the result for low n-6 LA was statistically significant. Overall, these findings support the current CVD prevention guidelines that recommend the consumption of foods rich in n-6 and n-3 PUFA to prevent CVD.^3,11^ For dietary recommendations, it is generally relevant to consider nutrient replacement; however, given their very low levels of intake (<1 g/day), biological effects of dietary EPA/DHA are not likely related to their replacing any specific nutrient. For LA, consumed at higher levels, replacement of saturated and trans fats has traditionally been recommended, although the scientific literature also suggests potential benefits of consuming LA in place of other macronutrients such as total carbohydrate and even monosaturated fats.

To our knowledge, no previous study has assessed interactions between circulating and adipose tissue fatty acids and family history of CVD in relation to the risk of CVD. However, a recent study by Zhang et al, based on the UK Biobank database, investigated interactions between self-reported dietary habits and family history of CVD in relation to the risk of future CVD.^12^ Among the specific dietary factors considered, total fish consumption was found not to interact with a family history of CVD. The intake of vegetable oils or nuts was not specifically studied. The study analyzed interactions solely using the product term approach. One possible reason for discrepancies between our results and those by Zhang et al^12^ may be that they used food frequency questionnaire data, whereas we used biomarkers. Further, the questionnaire they used did not separate questions about lean and oily fish, which may have hidden the beneficial effects of the n-3 EPA/DHA, which are found mainly in oily fish.

We speculate that our finding of interactions involving EPA/DHA may relate to specific cardiovascular related gene–diet interactions involving n-3 PUFA; such interactions have been proposed by other groups^44,45^ who performed genome-wide interaction studies in relation to CVD considering either fish oil supplementation^44^ or n-6 and n-3 PUFA biomarkers.^45^ In these studies, significant interactions with genes on the multiplicative scale were found for fish oil supplementation^44^ and the specific n-3 PUFA biomarkers ALA and DHA.^45^ No significant interaction between genes and n-6 PUFA biomarkers were identified.^45^ We further speculate that our observed significant interaction between low PUFA and family history of CVD indicates that in individuals with a predisposition to CVD, there is, to some extent, subclinical disease in which pathophysiological processes may be halted or reduced in the setting of higher endogenous levels of EPA/DHA. However, an alternative explanation for our finding could also be that individuals with low n-3 EPA/DHA may simultaneously have high concentrations of other fatty acids, for example trans fatty acids,^46^ which, through interaction with risk genes in individuals with a family history of CVD, could increase the risk of CVD. Yet another alternative explanation for our finding could relate to interactions with other factors that may cluster in families.

Our results from sensitivity analyses using the family history B definition generally support the main findings, although the interaction finding for low n-3 EPA/DHA was only borderline significant using the 25th percentile cut-off value and non-significant using the 50th percentile cut-off value. This is possibly attributable to the influence of chance, as the study sample was smaller. For family history definition A, the use of the 50th percentile PUFA cut-off value gave results that agree with the main findings.

### Strengths and Limitations

Our results were obtained using data from well-characterized cohorts included in a large established consortium, and the fact that we performed de novo individual-level analyses likely reduced publication bias.

An advantage of our study is that we used biomarkers of fatty acids, as opposed to self-reported dietary intake data which can help reduce measurement error and recall bias. In particular, biomarkers of PUFA including LA, EPA, and DHA have repeatedly shown good validation results compared with self-reported dietary intake.^47^ Detailed information on such validation studies based on cohorts included in the FORCE and forming part of the current meta-analysis is provided in Table S9. However, it is known that blood PUFA biomarkers, especially ALA, do not perfectly mirror the corresponding dietary fat intake because they are short term biomarkers that reflect the intake of fat during the previous days and weeks and also their concentrations are influenced by genetics, environmental factors and their internal metabolism.^48^ Furthermore, it may be that even the lowest cut-off (the 25th study-specific percentile) used to identify individuals with low PUFA may not capture low PUFA at the study level if the underlying study population has generally high intake of food containing PUFA, as seen for example in Japanese and Nordic Europeans.

A common challenge with studies of interaction, that is also present in our study, is interpreting results from interaction analyses performed with different approaches. Our findings showing absence of synergistic or antagonistic effects but still a significant potentiated risk of CVD in individuals with family history of CVD linked to low n-3 EPA/DHA must be interpreted with caution. Of note, discrepant findings, depending on analytic approach used, as in our study, are common when 2 exposures simultaneously under study have an effect on the outcome.^37^ It has been argued that the assessment of interaction on an additive scale is preferrable for answering public health–oriented research questions.^37^ At the same time, assessment of interaction both on a multiplicative and additive scale is recommended to broadly elucidate interaction issues.^37^

Despite the efforts made to harmonize data at study level, for some of our pooled results there was evidence of moderately high between-study heterogeneity, especially for the results that used family history definition B. This between-study heterogeneity may mainly have been driven by varying study population characteristics and varying definitions of family history of CVD across cohorts. The heterogeneity observed may also be due to differences regarding lipid compartment for measuring the fatty acids. However, results from analyses stratified by lipid compartment were similar to the main results.

Although we were able to harmonize the control for confounding across the different studies and have adjusted for many relevant covariates, it is possible that residual confounding is present, particularly considering that no adjustment was made for an overall healthy diet. However, the adjustments for fatty acid biomarkers, socioeconomic indicators, body mass index and lifestyle factors should to some extent account for diet.

Another study limitation is the potential misclassification of family history of CVD due to errors in self-reporting; it is unclear how this may affect the interaction estimates.^49^ However, we have used definitions of family history of CVD which are well-accepted and used in clinical practice.^3,10,50^

### Conclusions

The findings from this study suggest that low blood/tissue levels of n-3 EPA/DHA, reflecting a low intake of fats present in oily fish, may potentiate the risk of CVD in those already at increased risk because of family history. Low blood/tissue levels of n-6 LA and n-3 ALA, reflecting a low intake of fats present in vegetable oils and nuts, were not associated with amplification of CVD risk. Although these results should be interpreted with caution, it seems reasonable to conclude that our results support the current cardiovascular prevention guidelines regarding the consumption of foods rich in n-3 EPA/DHA (ie, oily fish), especially for people with a family history of CVD. Our side findings support the current recommendations stating that foods rich in n-6 LA and n-3 ALA such as vegetable oils and nuts should be a part of the diet.

## ARTICLE INFORMATION

### Acknowledgments

The authors thank Vikström Max for his support with statistical analyses. Study-specific acknowledgments are found in the Supplemental Material. Aggregate data used for the pooled analysis may be shared upon reasonable request. Each participating study may be able to share the original raw data on a selective case-by-case basis. Drs Laguzzi and Leander had full access to all the data in the study and take responsibility for the integrity of the data and the accuracy of the data analysis.

### Sources of Funding

This study received support from the Swedish Research Council (project 2019-01717 to K.L.) and the Swedish Heart Lung Foundation (project 20180540 to K.L.). Information about funding for each of the participating studies is available in the Supplemental Material.

### Disclosures

Dr Murphy reports having worked as a consultant for Pharmavite (until 2021). The remaining authors have reported no relationships relevant to the contents of this article. Dr Psaty serves on the steering committee of the Yale Open Data Access Project, funded by Johnson and Johnson.

### Supplemental Material

Supplemental Methods, Sources of Funding, and Acknowledgments

Tables S1–S9

Figures S1–S9

## Supplementary Material


