# Supplementary material for: Role of Polyunsaturated Fat in Modifying Cardiovascular Risk Associated With Family History of Cardiovascular Disease: Pooled De Novo Results From 15 Observational Studies
Source: Circulation. 2023 Dec 4;149(4):305–16. doi: 10.1161/CIRCULATIONAHA.123.065530 (PMC10798593; doi:10.1161/CIRCULATIONAHA.123.065530)
Supplement: Supplementary file 1 [file cir-149-305-s001.docx]

**SUPPLEMENTAL MATERIAL**

Table of contents:

Supplemental Methods page 1

Supplemental Tables S1-S9 page 2 - 25

Supplemental Figures S1-S9 page 26 - 34

Study specific funding information page 35 - 36

Study specific acknowledgements page 37

Supplemental Methods

The FORCE group is comprised of more than 100 experts worldwide in the field of fatty acids and health and represents over 60 cohorts. At regular scientific meetings every two weeks throughout the year, members review and provide feedback on proposed and ongoing projects - from inception to completion, including development of overall methodological approach, individual cohort statistical analysis, performance and interpretation of pooled results, manuscript drafting and critical review. Any investigator from the FORCE participating studies can propose projects and analysis amendments. After discussions at these meetings, the projects are also shared with the entire author group and eventually approved. For some participating studies, specific review and approval from cohort publication and/or coordinating committees is also needed. This procedure enables quality assurance of the projects carried out.

For the selection of covariates for use in analytic models to account for possible confounding, consideration is within the FORCE always given to the balance between what can be considered complete based on subject knowledge (highlighted as important in the STROBE guidelines, https://www.strobe-statement.org/) with what is practical and possible. Often, factors included in previous studies in the same field are also taken into account. For the current study, we considered factors included in previous studies of PUFA in relation to CVD risk, e.g., Del Gobbo et al. ^14^ and Marklund et al. ^19^, as well as previous research on interactions between PUFA and family history ^12^. We also considered the general complexity of confounding control in studies of interactions, which is primarily related to the need to control for confounding of the relationships between both factors with the outcome ^37^.

Table S1: Study-specific information

|  | **AGES-R**  **doi: 10.1093/aje/kwk115** |
| --- | --- |
| Full Name | Age, Gene/Environment Susceptibility-Reykjavik study |
| Sample source | Reykjavik Iceland |
| Description | The AGES-Reykjavik Study is a random sample of 5,764 men and women who were drawn from an established single center population based cohort; the Reykjavik Study, begun in 1967 to study heart disease. AGES-Reykjavik Study was designed to examine risk factors, including genetic susceptibility and gene/environment interaction, in relation to disease and disability in old age. At study baseline (2002–2006), participants were aged 66–96 years. A total of 753 adults with available data on circulating fatty acids and diabetes were eligible for the current analysis. |
| Ascertainment of fatty acid biomarker concentrations | Blood samples were collected at the AGES-Reykjavik baseline after an overnight fast and stored at -80C. Fatty acids were measured in plasma phospholipids at the Biomarker Laboratory, Fred Hutchinson Cancer Research Center. Plasma lipids were extracted by using the method of Folch. Phospholipids were separated from other lipids by using one-dimensional thin-layer chromatography. Fatty acid methyl esters were prepared by direct transesterification (19) and separated by using gas chromatography (Agilent Technologies 7890 Gas Chromatograph flame ionization detector; Supelco fused silica 100-m capillary column SP-2560; initially at 1608C for 16 min, ramped up at 3.08C/min to 2408C, and held for 15 min). The identification, precision, and accuracy were continuously evaluated by using both model mixtures of known fatty acid methyl esters and established in-house control pools. Fatty acids were expressed as the weight percentage of the total phospholipid fatty acids analyzed. The CV from pooled quality-control samples for LA, AA, ALA, EPA, DHA, and DPA were all ,2.5%. CVs for other major fatty acids were 0.77% (palmitic), 0.47% (stearic), and 0.42% (oleic). Ref: Harris TB, Song X, Reinders I et al. Plasma phospholipid fatty acids and fish-oil consumption in relation to osteoporotic fracture risk in older adults: the Age, Gene/Environment Susceptibility Study. Am J Clin Nutr 2015; 101(5):947-55. |
| Family history definition | The questionnaire asked whether participants had family history of hypertension, family history of stroke or family history of MI. No specifications were placed on 'family' or age of family members. |
| Missing data handling | Exclusion |
| CVD assessment | Total cardiovascular disease (CVD) (fatal or nonfatal myocardial infarction, coronary heart disease (CHD) death, sudden cardiac death, or ischemic stroke determined by self-report and medical records |
|  | **KIHD**  **Ann Clin Res. 1988;20(1-2):46-50** |
| Full Name | The Kuopio Ischaemic Heart Disease Risk Factor Study (KIHD) |
| Sample source | Eastern Finland |
| Description | The KIHD is a prospective, population-based cohort study that was designed to investigate risk factors for CVD, atherosclerosis, and related outcomes in a population-based sample of men from eastern Finland. The baseline examinations were carried out in 1984-1989. A total of 2682 men who were 42, 48, 54 or 60 years old at baseline (82.9% of those eligible) were recruited in two cohorts. The first cohort consisted of 1166 men who were 54 years old, enrolled in 1984-1986, and the second cohort included 1516 men who were 42, 48, 54 or 60 years old, enrolled in 1986-1989. |
| Ascertainment of fatty acid biomarker concentrations | Venous blood samples were collected between 8AM and 10AM after an overnight fast. Serum total fatty acids were determined from frozen samples with a NB-351 capillary column (HNU-Nordion, Helsinki, Finland) by a Hewlett-Packard 5890 Series II gas chromatograph (Hewlett-Packard Company, Avondale, Pa, USA, since 1999 Agilent Technologies Inc., USA) with a flame ionization detector. Serum was extracted with chloroform-methanol and fatty acids were methylated with methanol and sulphuric acid prior to gas chromatography. Each analyte had an individual reference standard and the analytes were quantified with an internal standard method using eicosane. The coefficient of variation (CV%) for repeated measurements of fatty acids was 5.8% for alpha-linolenic acid (ALA, 18:3n-3), 5.9% for eicosapentaenoic acid (EPA, 20:5n-3), 5.7% for docosahexaenoic acid (DHA, 22:6n-3), 9.9% for arachidonic acid (ARA, 20:4n-6) and 8.7% for linoleic acid (LA, 18:6n-2). |
| Family history definition | Family history of CVD was determined from paper questionnaires and verified by a study nurse during the baseline study visit. Individuals who reported having one or more close relatives (mother, father and siblings considered) affected by either a clinically diagnosed heart attack or stroke with a fatal or non-fatal outcome were considered to have a family history of CVD under definition A. There is no information whether only full siblings should be regarded. |
| Missing data handling | For missing covariate data, a missing indicator category was used for categorical covariates. Missing continuous covariates were replaced with the cohort mean. |
| CVD assessment | The follow-up for these analyses extended until Dec 31, 2012. Deaths were ascertained by linkage to the national Causes of Death Register using the personal identification codes (social security number). Data on fatal and non-fatal coronary events were obtained by computer linkage to the national hospital discharge and death certificate registers. The diagnostic classification of events was based on symptoms, electrocardiographic (ECG) findings, cardiac enzyme elevations, autopsy findings (80%), and history of CHD together with the clinical and ECG findings of the paramedic staff. All the documents were cross-checked in detail by two physicians. Diagnostic information was collected from hospitals and classified using identical diagnostic criteria. Each suspected coronary event (ICD-9 codes 410–414 and ICD-10 codes I20–I25) was classified into a definite acute MI, a probable acute MI, a typical acute chest pain episode of more than 20 min indicating CHD, an ischemic cardiac arrest with successful resuscitation, or no acute coronary event by a physician using the original patient records. Acute coronary events that did not lead to death during the following 24 hours were considered as a non-fatal event. Incident strokes between the years 1984-1992 were observed through FINMONICA stroke register and between years 1993 and 2012 through computerized linkage to the national hospital discharge registry. The diagnosis of stroke was based on sudden onset of clinical signs or focal or global disturbance of cerebral function lasting 24 hours (except in the case of sudden death or if interrupted by surgical intervention) with no apparent cause other than a vascular origin. Each suspected stroke (International Classification of Diseases [ICD]-9 codes 430-439 and ICD-10 codes I60–I68 and G45–G46) was classified into 1) a definite stroke, 2) no stroke, or 3) an unclassifiable event. The FINMONICA stroke register data were annually rechecked with the data obtained from the computerized national hospital discharge and death registers. Definite strokes and unclassifiable events were included in the group of any stroke. Each definite stroke was classified into 1) an ischemic stroke (ICD-9 codes 433-434; ICD-10 code I63) or 2) a hemorrhagic stroke (ICD-9 codes 430-431; ICD-10 codes I60-I61). If the subject had multiple nonfatal strokes during follow-up, the first stroke was considered as the end point. CT was performed in 90% of the patients by 1993, and CT, MRI, and autopsy reached 100% by 1997. |
|  | **MESA**  **doi: 10.1093/aje/kwf113** |
| Full Name | Multi-Ethnic Study of Atherosclerosis (MESA) |
| Sample source | 6 U.S. communities: Wake Forest (NC), UCLA (CA), Northwestern University (IL), University of Minnesota (MN), Columbia (NY), Johns Hopkins (MD) |
| Description | The Multi-Ethnic Study of Atherosclerosis (MESA) is a study of the characteristics of subclinical cardiovascular disease (disease detected non-invasively before it has produced clinical signs and symptoms) and the risk factors that predict progression to clinically overt cardiovascular disease or progression of the subclinical disease. MESA researchers study a diverse, population-based sample of 6,814 individuals free from overt signs of CVD at baseline, of European- African- Hispanic- and Chinese- ancestry ascertained across six field centers across the United States. Baseline data for the current analyses were taken from the first clinic exam conducted in 2000 – 2002. In addition to yearly phone calls, follow-up clinic exams are conducted approximately every two years, and at the time the current analyses were conducted incident CVD was available until the fifth clinic exam conducted in 2010-2011. The sample included in this study was composed of those 2,262 participants who had available fatty acid and follow-up for CVD. |
| Ascertainment of fatty acid biomarker concentrations | Fatty acids were measured in EDTA plasma frozen at -70˚C using samples collected after a 12-hour fast. Plasma phospholipids were isolated by thin layer chromatography, with FAs being subsequently separated by gas chromatography. The Collaborative Studies Clinical Laboratory at Fairview-University Medical Center (Minneapolis, MN) performed the FA assays. Individual FAs were expressed as a percentage of total FAs. |
| Family history definition | Family history of heart attack (parents/siblings/children) |
| Missing data handling | There were no missing covariates. |
| CVD assessment | Incident cardiovascular disease events |
|  | **EPIC-Norfolk**  **doi: 10.1371/journal.pmed.1001255** |
| Full Name | European Prospective Investigation into Cancer and Nutrition Norfolk study (EPIC-Norfolk Study) |
| Sample source | Norfolk, England |
| Description | The European Prospective Investigation into Cancer (EPIC)-Norfolk is a prospective study of 25,639 men and women aged 40–79 years in Norfolk, UK similar in characteristics to UK general population samples, who participated in a baseline survey in 1993–1997. Participants completed a health and lifestyle questionnaire including data on medical history, smoking, alcohol intake, physical activity, social class, and education and attended a clinic for a health examination. Body mass index (BMI) was calculated as weight (kilograms) divided by height (meters) squared. Blood pressure was measured using an Accutorr non-invasive blood pressure monitor. Blood samples were spun, separated into 0.5 ml fractions of serum and citrated plasma, placed in straws, sealed, and stored in liquid nitrogen. |
| Ascertainment of fatty acid biomarker concentrations | Funding was obtained for blood FA analyses in 2003–2008. Selection of participants for analyses was based on a series of nested case control studies with incident cases of cancers and cardiovascular disease and up to four disease-free controls for each case. Citrated plasma straws were retrieved from liquid nitrogen storage, thawed at room temperature and 20 mg of di-palmitoyl-D31-phosphatidylcholine (Sigma, St. Louis, MO) internal standard was added to each 200 ml plasma sample. Following extraction of total lipids with chloroform/methanol, phospholipids were further purified by adsorption chromatography (LC-Si SPE, Supelco/Sigma, St. Louis, MO), trans methylated to fatty acid methyl esters and extracted with hexane. Analysis was carried out by gas chromatography with flame ionization detection (220°C) using a 30 m x 0.32 mm x 0.2 um SP2340 fused silica capillary column (Supelco/Sigma, St. Louis, MO). Carrier gas was Helium at a constant flow of 1.3 ml/min. Samples of 0.5 µl were introduced onto the column via on-column injection. The column was held initially for 1 min at 65°C, then programmed at 5°C/min to 135°C, then at 2°C/min to 200°C, and finally at 10°C/min to 220°C. Run time was 60 min. Identification of individual fatty acid methyl esters was based on comparison with retention times of authentic standards (Sigma, St. Louis, MO). Plasma concentrations were measured by comparison of peak areas of individual fatty acids with the peak area of the palmitoyl-D31-fatty acid methyl ester internal standard using individual calibration curves for each of the 22 fatty acid methyl esters measured. The chromatographic peak for palmitoyl-D31-fatty acid methyl ester elutes about 1 minute earlier than non-labelled palmitoyl fatty acid methyl ester in a zone free of interference from other peaks. Each chromatogram was integrated automatically and checked for accuracy and specificity by a laboratory technician. Results were reported as mol% for each individual FA, considering the large difference in molecular mass between the shortest (myristic acid, 14:0) and longest (docosapentaenoic acid, 22:5n-3) chain FA measured. Analytical quality control was carried out by the daily use of standard quality control plasma samples. The CVs for the major fatty acids were between 3% and 13%. |
| Family history definition | A questionnaire on general health characteristics was administered to ask family history for noncommunicable diseases including "heart attack" and "stroke" in first degree relatives. Family history for cardiovascular diseases was defined as an affirmative response about heart attack, stroke, or both. |
| Missing data handling | For missing covariate data, a missing indicator category was used for categorical covariates. Missing continuous covariates were handled by exclusion from the analysis. |
| CVD assessment | All participants are flagged for death certification with the National Health Service Central Register, UK with death certificates coded by nosologists according to the International Classification of Disease (ICD). Deaths due to coronary heart disease were defined using ICD9 410-414 or ICD10 I20-I25 as underlying cause of death; and deaths due to stroke, ICD9 430-438 or ICD10 I60-I69. Deaths due to cardiovascular diseases were defined as a death due to either coronary heart disease or stroke. The mortality data up to 30 June 2013 were collected. For incidence data, the East Norfolk Health Authority database was used to identify all hospital contacts for participants using their National Health Service number. We used the ICD diagnostic codes listed to ascertain hospital episodes for coronary heart disease and stroke. Participants were identified as having an event during follow-up if they had a hospital admission and/or died with each episode as cause of death, with clinical validation through medical record inspection of a sample.18 We ascertained the events up to December 2009. |
|  | **CHS**  **doi: 10.1016/1047-2797(91)90005-w** |
| Full Name | Cardiovascular Health Study (CHS) |
| Sample source | 4 U.S. communities: Forsyth County, North Carolina; Sacramento County, California; Washington County, Maryland; Allegheny County, Pennsylvania |
| Description | The CHS Study is a prospective population-based cohort study of people ≥ 65 years old at baseline initiated to evaluate risk factors for the development and progression of cardiovascular disease. Participants were recruited at four field centers (Forsyth County, NC; Sacramento County, CA; Washington County, MD; Pittsburgh, PA) from random samples of Medicare eligibility lists. The cohort consists of 5201 non-institutionalized men and women, recruited in 1989-1990, plus an additional 687 predominately black participants recruited in 1992-93. A total of 2644 adults with available data on circulating fatty acids and sibling history of CVD and without prior CVD were eligible for the current analysis. |
| Ascertainment of fatty acid biomarker concentrations | Plasma phospholipid fatty acids were measured at the Fred Hutchinson Cancer Research Center (Seattle, WA) using stored blood samples from 1992-1993. Total lipids were extracted from plasma using the methods of Folch. A one dimensional thin-layer chromatography was used to separate phospholipids from neutral lipids. Phospholipids fraction was directly trans-esterified using the Lepage and Roy method to prepare fatty acid methyl esters, and individual fatty acid methyl esters were separated using gas chromatography (Agilent 5890 Gas Chromatograph flame ionization detector, Agilent Technologies, Palo Alto, CA; fused silica capillary column SP-2560 [100m x 0.25mm, 0.2μm], Supelco Belefonte, PA; initial 160 degrees Celsius for 16 min, ramp 3 degrees Celsius/min to 240 degrees Celsius, hold 15 minutes). All fatty acids were processed at the Biomarker Laboratory of the Fred Hutchinson Cancer Research Center (Seattle, WA). For this analysis, levels of each individual fatty acid including 20:0, 22:0, and 24:0 are expressed as a weight percentage of total phospholipid fatty acids analyzed. Inter-assay coefficient of variations for the VLSFA measurements were ≤ 3.5%. |
| Family history definition | Sibling history of heart attack or stroke was determined at baseline on paper questionnaires (1989-1990 for cohort 1 and 1992-1993 for cohort 2). They were asked to write the names of all siblings and circle their gender. Additional information recorded that is used in this analysis includes whether they ever had a heart attack, age at first heart attack, whether they ever had a stroke, and age at first stroke. Note, information on history of CVD was not obtained for parents. Directions did not indicate whether only full siblings should be regarded but did specify to include those who have died or with whom the participant has lost touch. |
| Missing data handling | For missing covariate data, a missing indicator category was used for categorical covariates. Missing continuous covariates were handled by exclusion from the analysis except for BMI and smoking status. Smoking status was carried forward from prior years and height was carried forward from cohort 1 baseline to calculate BMI if height but not weight was missing; if after carry-forward, these were still missing, they were excluded from the analysis. |
| CVD assessment | CVD was defined as probable or definite MI, angina, angioplasty, bypass surgery or ischemic stroke, or cause of death recorded as Atherosclerotic CHD. Events were adjudicated by a panel of doctors and incident events are through June 30, 2015. Individuals with CVD prior to the 1992-1993 exam were excluded from the analysis. |
|  | **CRHS**  **doi: 10.1161/01.CIR.0000058165.81208.C6** |
| Full Name | Costa Rica Heart Study (CRHS) |
| Sample source | Costa Rica |
| Description | The CRHS is a matched case control study of non-fatal myocardial infarction conducted from 1994 to 2004. Survivors of a first MI by 2 independent cardiologists at any of the 6 recruiting hospitals in the catchment area were identified as eligible cases. All cases met the World Health Organization criteria for MI at the time of recruitment, which require typical symptoms plus either elevations in cardiac enzyme levels or diagnostic changes in EKG. Cases were ineligible if they died during hospitalization, were ≥ 75 years old on the day of their first MI or were physically or mentally unable to answer the questionnaire. For each case, one population control was selected randomly with the aid of data available from the National Census and Statistics Bureau of Costa Rica, matched by age (± 5 y), sex, and area of residence. |
| Ascertainment of fatty acid biomarker concentrations | [Fatty acids from adipose tissue were quantified by gas-liquid chromatography. Peak retention times and area percentages of total fatty acids were identified by injecting known standards (NuCheck Prep) and analyzed with Agilent Technologies ChemStation A.08.03 software. Twelve duplicate samples, indistinguishable from the others, were analyzed throughout the study. The coefficients of variation for EPA, DHA, α-linolenic acid, and linoleic acid were 20.2%, 14.7%, 6.4%, and 3.6%, respectively.](https://www.ahajournals.org/doi/10.1161/01.cir.0000058165.81208.c6#R17-124046) |
| Family history definition | Family history of myocardial infarction was determined by interview. Participants were asked if any of their first-degree relatives (father, mother and siblings) had or died from myocardial infarction before 60 years old (for both males and females). Whether siblings had to be full siblings was not specified. |
| Missing data handling | Missing values were excluded except for the income variable that was handled with the indicator method. |
| CVD assessment | Incident cases of a first non-fatal myocardial infarction were recruited from the 6 main hospitals in the central valley of Costa Rica from 1994 to 2004. All cases met the World Health Organization criteria for MI at the time of recruitment, which require typical symptoms plus either elevations in cardiac enzyme levels or diagnostic changes in EKG. Cases were ineligible if they died during hospitalization, were ≥ 75 years old on the day of their first MI or were physically or mentally unable to answer the questionnaire. |
|  | **MCCS**  **doi: 10.1093/ije/dyx085** |
| Full Name | Melbourne Collaborative Cohort Study (MCCS) |
| Sample source | Melbourne, Victoria, Australia |
| Description | "The Melbourne Collaborative Cohort Study (MCCS) is a prospective cohort study of 41,513 women and men aged 27 to 75 years (99% were between 40-69 years) when recruited between 1990 and 1994 [Milne et al. Int J Epidemiol, 2017]. Italian and Greek migrants were over-sampled to extend the range of lifestyle exposures. Participants were recruited via the electoral rolls (registration to vote is compulsory for adults in Australia), advertisements, and community announcements in local media (e.g. television, radio, and newspapers). Comprehensive lists of Italian and Greek surnames in the phone book and Electoral Rolls were also used to target southern European migrants. The Cancer Council Victoria’s Human Research Ethics Committee approved the study protocol. Participants gave written consent to participate and for the investigators to obtain access to their medical records. Vital status and cause of death information were obtained via linkage to the National Death Index of Australia.  A case-cohort design was used to measure plasma fatty acids using baseline blood samples. The case-cohort study included all participants who had died from cardiovascular disease between baseline and 30 June 2002 (n = 532) and a random sample (sub-cohort) of all MCCS participants (n = 4659, which included 51 CVD deaths). After excluding 175 CVD deaths that were not due to CHD or stroke, and excluding those with no fatty acids data (N=180), missing data for family history of CVD (N=66), participants with self-reported CHD or stroke at baseline (N=382) and also excluding 21 people with missing data for dyslipidemia and fish/cod liver oil supplements (N=21), there were 4316 participants (with 185 CVD deaths) eligible for the current analysis. |
| Ascertainment of fatty acid biomarker concentrations | Total lipids were extracted from plasma with chloroform/methanol (2:1, by volume). Lipid extracts were separated by thin-layer chromatography (TLC) into PL, triglyceride and CE classes on silica gel plates (Silica gel 60H Merck Darmstadt Germany). The TLC solvent system was petroleum spirit:diethyl ether:glacial acetic acid (180:30:2, by volume). Lipid classes were visualized with Fluorescein 5-Isothiocyanate against TLC standard 18-5 (NuChek Prep Inc: Elysian, MN). All solvents contained the anti-oxidant butylated hydroxy anisole at 0.005% (wt/vol). Phospholipid fractions were trans-esterified by methanolysis (1% H2SO4 in methanol) for three hours at 70oC. After cooling, the resulting fatty acid methyl esters (FAME) were extracted with n-heptane and transferred into gas chromatography vials containing anhydrous Na2SO4. FAME were separated and quantified with a Hewlett-Packard 5880 gas-liquid chromatograph using a capillary column equipped with flame ionization detection and Hewlett-Packard Chem-Station data system. Separation was achieved on a 50m x 0.33mm ID. BPX-70 column (SGE, Melbourne, Australia). Helium was the carrier gas at a column flow rate of 35 cm per second. The inlet split ratio was set at 30 to 1. The oven temperature at injection was set at 140oC and programmed to rise to 220oC at 5oC per minute. The injector and detector temperatures were set at 250oC and 300oC, respectively. FAME were identified by comparison of retention times to authentic lipid standards (NuChek Prep Inc: Elysian, MN).  The coefficients of variation were between 2%-15%: PPL%  ** eicosapentaenoic acid [EPA] 20:5n3CV 6%  ** docosahexaenoic acid [DHA] 22:6n3 CV 5%  ** linoleic acid [LA] 18:2n6 CV 2%  ** alpha linolenic acid [ALA] 18:3n3 CV 15%  ** arachidonic acid [AA] 20:4n6 CV 3%" |
| Family history definition | Family history of CVD was determined from interviewer-administered questionnaires at baseline (1990-94). Participants were asked whether their mother or father had "ever had a heart attack" or whether they had "ever had a stroke". Participants who reported they had at least one brother (or one sister) were asked whether their sibling(s) had ever had a heart attack, or whether they had ever had a stroke. The questionnaire did not specify whether reported diseases in close relatives needed to be clinically diagnosed. Whether siblings had to be full siblings was not specified. |
| Missing data handling | Missing continuous covariates were handled by exclusion from the analysis (alcohol in g/d, BMI). For the categorical covariates included in the models, only 2 other variables had missing data: dyslipidemia (N=6) and fish/cod liver oil supplements (N=15). These were excluded as there were not many people in the missing categories for these, and this created issues because dyslipidemia has to be included in the strata option of the Cox model in Stata to deal with non-proportional hazards. |
| CVD assessment | In the MCCS, deaths were identified by at least annual linkage to the Victorian Registry of Births, Deaths and Marriages (considered complete) and by at least 2-yearly record linkage to the National Death Index (NDI), compiled by the Australian Institute of Health and Welfare (to which all state and territory death registries contribute). Linked NDI data also include cause of death. For the case-cohort study, participants were followed up from baseline until 30th June 2002 (or date of death or migration from Australia, which ever came first). During the follow-up (median 9 years), 125 men and 60 women died from CVD. Participants that had a prior event of heart attack, angina, or stroke (self-reported) at baseline were excluded by analysis (n=407). |
|  | **METSIM**  **doi: 10.3945/ajcn.113.069740, 10.1007/s00125-015-3730-5, 10.1194/jlr.O072629** |
| Full Name | The Metabolic Syndrome in Men (METSIM) |
| Sample source | Kuopio town, Eastern Finland, Finland |
| Description | The METSIM study includes 10,197 men, aged from 45 to 73 years at entry, randomly selected from the population register of the Kuopio town, Eastern Finland, and examined in 2005–2010 (3). The aim of the study is to investigate nongenetic and genetic factors associated with T2D and CVD, and with cardiovascular risk factors in both cross-sectional and longitudinal settings. Fatty acid composition of erythrocyte membranes and plasma lipids was available in 1364 participants at baseline. |
| Ascertainment of fatty acid biomarker concentrations | Erythrocyte membranes: Erythrocytes were separated from EDTA-blood and then hemolyzed in the tris-HCl buffer (pH 7.6, 10 mmol/L). Fatty acid methyl esters were prepared by direct trans-esterification using acetyl chloride and analyzed by 7890A gas-chromatograph (Agilent Technologies, Inc., Wilmington, DE, USA) equipped with a 25-m free FA phase column (Agilent Technologies). Pure standards (NU Chek Prep Inc) were used to identify FA methyl esters and to prepare calibration curves. Heptadecanoic acid methyl ester (17:0) served as an internal standard. Intra-assay CVs for erythrocyte membrane 18:2n-6, 18:3n-3, 20:5n-3 and 22:6n-3 were 0.49, 2.27, 1.07 and 1.82 %, respectively. Phospholipids and cholesterylesters: Lipids were extracted from plasma sample with chloroform-methanol (2:1) and lipid fractions were separated with an aminopropyl column. FAs in lipid fractions were trans methylated with 14% boron trifluoride in methanol. Finally, FA methyl esters were analyzed by 7890A gas-chromatograph (Agilent Technologies, Inc., Wilmington, DE, USA) equipped with a 25-m free FA phase column (Agilent Technologies). Cholesteryl nonadecanoate (Nu Chek Prep, Inc., Elysian, MA, USA), and phosphatidylcholine dinonadecanoyl (Larodan Fine Chemicals, Malmo, Sweden) served as internal standards.The intra-assay CVs for phospholipid 18:2n-6, 18:3n-3, 20:5n-3 and 22:6n-3 were 0.6, 3.6, 0.6 and 0.7 %, respectively and for cholesteryl esters 18:2n-6, 18:3n-3, 20:5n-3 and 22:6n-3 were 0.1, 0.3, 0.5 and 0.7, respectively. |
| Family history definition | Family history of CVD at baseline was determined based on the following question. Do you have early coronary heart disease among family members or relatives (<55 years men, <65 years women)?  1. NO  2. YES: grandparents, parents’ siblings or cousins but NOT own parents, siblings or children  3. YES: own parents, siblings or children  Definition B was used by recoding variable so that 1 and 2 are no family history of CVD, and 3 is yes. |
| Missing data handling | There were no missing covariates. |
| CVD assessment | Total myocardial infarction event or coronary artery disease death or cerebral infarction events by 2018-12-13. Participants that had a prior event of CVD at the baseline were excluded by analysis (n=10). |
|  | **EPIC-Potsdam**  **doi: 10.1159/000012787** |
| Full Name | European Prospective Investigation into Cancer and Nutrition Potsdam study (EPIC-Potsdam Study) |
| Sample source | Potsdam and surroundings, Germany |
| Description | The European Prospective Investigation into Cancer and Nutrition (EPIC)-Potsdam Study is part of the multi-centre prospective cohort study EPIC. In Potsdam, Germany, 27,548 subjects (16,644 women aged mainly 35-65 years and 10,904 men aged mainly 40-65 years) from the general population were recruited between 1994 and 1998. We randomly selected 2,500 individuals from all participants of the EPIC-Potsdam study population who provided blood samples (n=26,444) for a sub-cohort. |
| Ascertainment of fatty acid biomarker concentrations | Thirty milliliters of blood were taken from each participant during baseline examination and were centrifuged at 1000 g for 10 min at 4°C. Plasma, serum, red blood cells, and buffy coat were removed and stored at –80°C. The erythrocyte membrane fatty acids (FA) were analyzed at the Laboratory of the Dutch National Institute for Public Health and Environment between February and June 2008. Briefly, FA methyl esters (FAME) were separated on a GC-3900 gas chromatograph (Varian Inc., Middelburg, Netherlands) equipped with a 100 m x 0.25mm ID WCOT-fused silica capillary column and flame ionization detector with separation of FAME peaks based on mixed FAME standards (Sigma Aldrich, St Louis, USA). The Galaxie software version 1.9.3.2 (Varian Inc.) was used for quantification and identification of peaks. The FAs were expressed as the percentage of total FAs present in the chromatogram. Intraassay CVs (%): LA: 2.2, ALA: 10.2, EPA: 3.1, DHA: 2.4 |
| Family history definition | Family history of CVD was determined from questions in paper questionnaires distributed to the participants at 5th follow-up. Individuals who reported having one or more close relatives (mother, father and siblings considered) affected by either myocardial infarction or stroke were considered to have a family history of CVD under definition A. Individuals who reported that a close relative was affected by MI or stroke before he or she turned 60 were considered to have a family history under definition B. All information on family history relies on self-reported data and refers to genetic parents and full siblings. |
| Missing data handling | For missing covariate data, a missing indicator category was used for categorical covariates. Missing continuous covariates were handled by exclusion from the analysis. |
| CVD assessment | About every 2 years, information on incident diseases was collected. To identify potential CVD cases, several sources were used: self-report, death certificate or linkage with hospital information system. All identified potential CVD events were ascertained by study physicians, in cooperation with the patients’ attending physicians and hospitals, who provided a detailed medical verification of self-reports and death certificates by clinical records. Total CVD was defined as fatal or nonfatal myocardial infarction (MI), sudden cardiac death or ischemic stroke. We considered data until the end of the fifth follow-up period (year 2009). Participants that had prevalent MI, stroke or angina pectoris were excluded from analysis (n=211). |
|  | **FHS**  **doi: 10.1093/oxfordjournals.aje.a112813** |
| Full Name | Framingham Heart study - Offspring cohort |
| Sample source | Framingham, MA, USA |
| Description | Our analysis focused on the Framingham Heart Study (FHS) Offspring sample, a population based longitudinal study of families living in Framingham, Massachusetts. The offspring study was initiated in 1971 and consisted of a sample of 5,124 individuals, offspring of the original cohort and their spouses. |
| Ascertainment of fatty acid biomarker concentrations | The fatty acid composition of RBC samples was analyzed by gas chromatography equipped with a SP 2560 capillary column after direct transesterification for 10 minutes in boron trifluoride/ methanol and hexane at 100 C. This technique generates fatty acids primarily from RBC glycerophospholipids. RBCs were isolated from blood drawn after a 10–12 h fast and frozen at −80 °C immediately after collection. All fatty acids present at >1% abundance had CVs of ≤7%. |
| Family history definition | For most individuals, a family history of CVD was determined via adjudicated outcomes in the same manner as primary outcome assessment. This is because the primary analysis focused on the Framingham "Offspring" cohort and their parents (and siblings) were also in the Framingham Heart Study. Thus, detailed information on age of onset and precise CVD definitions (see below) were used. However, in ~876 situations adjudicated outcomes were not available for the father and in 849 cases adjudicated outcomes were not available for mothers. In these cases, self-reported family history was used. In particular, self-report about (a) MI before aged 55 (mother or father), (b) CABG before age 55 (mother or father), (c) stroke before age 65 or (d) death from CVD was used for Definition A and definition B used (a), (b) and (c) (but not (d)). Sibling CVD was adjudicated for all participants on at least one sibling, but no self-report was available for siblings not in the study. |
| Missing data handling | For missing covariate data, a missing indicator category was used for categorical covariates. Missing continuous covariates were handled by exclusion from the analysis. |
| CVD assessment | CVD events are ascertained annually, with data through 2014 available for this analysis. All identified events are adjudicated by the FHS Endpoint review committee and represent a combination of both active and passive ascertainment. In particular, information about CVD events on follow-up was obtained with the aid of medical histories, physical examinations at the study clinic, hospitalization records, and communication with personal physicians. |
|  | **Hisayama**  **doi: 10.2337/dc12-0166, 10.1016/j.atherosclerosis.2013.09.023** |
| Full Name | The Hisayama Study |
| Sample source | Hisayama town, Kasuya-gun, Fukuoka prefecture, Japan |
| Description | The Hisayama Study is an ongoing, population-based prospective cohort study of cardiovascular disease and its risk factors in the town of Hisayama, a suburb in the metropolitan in Japan. A total of 3,103 residents who were aged 40 years older, without cardiovascular disease at baseline, and had no missing values for serum fatty acid levels were enrolled in the present study. |
| Ascertainment of fatty acid biomarker concentrations | Serum fatty acids levels were assayed by gas chromatography (SRL, Tokyo, Japan). Briefly, total lipids in plasma were extracted according to the Folch’s procedure, followed by hydrolysis to free fatty acids. Free fatty acids were esterified with potassium methoxide/methanol and boron trifluorideemethanol. The methylated fatty acids were analyzed using GC-17A gas chromatograph (Shimadzu Corporation, Kyoto, Japan) with omegawax-250 capillary column (SUPELCO, Sigma Aldrich Japan, Tokyo, Japan). Reproducibility (i.e., the coefficient of variation) of the determination of serum EPA, DHA, and AA levels by this method was reported to be 4.4%, 2.3%, and 3.8%, respectively. |
| Family history definition | Family history of CVD was determined from self-reported questionnaires distributed to the participants, which conformed at the baseline survey by research nurses. Individuals who reported having one or more close relatives (mother, father, siblings and child) affected by either myocardial infarction or stroke were considered to have a family history of CVD under definition A. Definition B could not be determined as age of the onset of CVD among family members were not available. The questionnaire did not specify whether reported diseases in close relatives needed to be clinically diagnosed. Although, whether siblings had to be full siblings was not specified; we usually consider siblings as full siblings in Japan. |
| Missing data handling | For missing covariate data, a missing indicator category was used for categorical covariates. Missing continuous covariates were handled by exclusion from the analysis. |
| CVD assessment | Cardiovascular disease was defined as first-ever development of stroke or coronary heart disease. The diagnosis of stroke and coronary heart disease were based on the clinical history, physical examination (including neurological examination) and/or all available clinical record and data, including brain CT/MRI and autopsy finding. Stroke was defined as a sudden onset of nonconvulsive and focal neurological deficit persisting for >24 h. The criteria for a diagnosis of coronary heart disease included first-ever acute myocardial infarction, silent myocardial infarction, sudden cardiac death within 1h after the onset of acute illness, or coronary artery disease followed by coronary artery bypass surgery or angioplasty. Acute myocardial infarction was diagnosed when a subject met at least two of the following criteria: (1) typical symptoms, including prolonged severe anterior chest pain; (2) abnormal cardiac enzymes more than twice the upper limit of the normal range; (3) evolving diagnostic electrocardiographic changes; and (4) morphological changes, including local asynergy of cardiac wall motion on echocardiography, persistent perfusion defect on cardiac scintigraphy, or myocardial necrosis or scars >1 cm long accompanied by coronary atherosclerosis at autopsy. Silent myocardial infarction was defined as myocardial scarring without any historical indication of clinical symptoms or abnormal cardiac enzyme changes, and was detected by electrocardiography, echocardiography, cardiac scintigraphy, or autopsy. |
|  | **InCHIANTI**  **doi: 10.1111/j.1532-5415.2000.tb03873.x** |
| Full Name | Invecchiare in Chianti |
| Sample source | Tuscany, Italy |
| Description | The InCHIANTI study is a population-based epidemiological study aimed at evaluating the factors that influence mobility in the older population living in the Chianti region in Tuscany, Italy. 1616 residents were selected from the population registry of Greve in Chianti (a rural area: 11,709 residents with 19.3% of the population greater than 65 years of age), and Bagno a Ripoli (Antella village near Florence; 4,704 inhabitants, with 20.3% greater than 65 years of age). The participation rate was 90% (n=1453), and the subjects ranged between 21-102 years of age. Following the baseline visit between 1998-2000, there were four follow up visits at 2001-2003, 2004-2006, 2007-2009, and 2013-2014. |
| Ascertainment of fatty acid biomarker concentrations | Plasma fatty acids measurement method has been described previously (PMID 16234304). Briefly, blood samples were collected in the morning after a 12-hr overnight fast. Aliquots of plasma were immediately obtained and stored at -80 C. Fatty acid methyl esters (FAME) were prepared through transesterification using Lepage and Roy’s method with modification Rodriguez-Palmero et al (PMID 13428781, 9474792). Separation of FAME was carried out on an HP-6890 gas chromatograph (Hewlett- Packard, Palo Alto, CA) with a 30-m fused silica column (HP-225; Hewlett-Packard). FAMEs were identified by comparison with pure standards (NU Chek Prep, Inc., Elysian, MA). For quantitative analysis of fatty acids as methyl esters, calibration curves for FAME (ranging from C14:0 to C24:1) were prepared by adding six increasing amounts of individual FAME standards to the same amount of internal standard (C17:0; 50xg). The correlation coefficients for the calibration curves of fatty acids were in all cases higher than 0.998 in the range of concentrations studied. Fatty acid concentration was expressed as a percentage of total fatty acids. The coefficient of variation for all fatty acids was on average 1.6% for intraassay and 3.3% for interassay. |
| Family history definition | Family history of CVD was determined using a questionnaire administrated by a trained interviewer. Participants with first degree relative (father, mother, or siblings) affected by myocardial infarction, angina, or stroke were considered to have a family history of CVD under definition A. |
| Missing data handling | Participants with missing data on main outcome and exposures were excluded from the study. |
| CVD assessment | Incident cardiovascular disease (MI, angina, stroke, CHF) was self-reported during the structured medical interview by trained staff at each visit. |
|  | **WHIMS**  **doi: 10.1001/jama.291.24.2959, doi: 10.1016/s0197-2456(98)00038-5** |
| Full Name | Women's Health Initiative Memory Study |
| Sample source | 50 participating centers |
| Description | WHIMS randomized trials which examined the effects of postmenopausal hormone therapy on cognitive function in women aged 65-80 years. Recruitment began in 1995. |
| Ascertainment of fatty acid biomarker concentrations | The fatty acid composition of RBC samples was analyzed by gas chromatography equipped with a SP 2560 capillary column after direct transesterification for 10 minutes in boron trifluoride/ methanol and hexane at 100 C. This technique generates fatty acids primarily from RBC glycerophospholipids. During the aliquoting phase, the RBC samples were stored improperly at -20°C for a period of approximately 2 weeks, causing oxidative degeneration of the PUFAs before measurement. The original FA levels were estimated with multiple imputations using independent data on fatty acid degradation and length of time the samples were exposed to -20°C [Pottala et al. 2012]. All fatty acids present at >1% abundance had CVs of ≤6.5%. Pottala JV, Espeland MA, Polreis J, Robinson J, Harris WS (2012) “Correcting the effects of -20°C storage and aliquot size on erythrocyte fatty acid content in the Women’s Health Initiative” Lipids. 47(9):835-46. |
| Family history definition | For family history of CVD, stroke and MI were combined for Definition A. For Definition B, we only used history of MI since we did not have age information on familial stroke. A cutoff of 65 years old was used for both men and women for Def B. The survey did not indicate only full siblings. |
| Missing data handling | Exclusion for quantitative variables; indicator for missing data on categorical covariates |
| CVD assessment | Defined as the first occurrence of clinical CHD or stroke or possible CHD or stroke. Clinical CHD and stroke were adjudicated for CT and OS ppts through Ext1. |
|  | **CIRCS**  **doi: 10.2188/jea.JE20180196, doi: 10.1253/circj.CJ-18-0240, 10.1161/01.str.0000023890.25066.50** |
| Full Name | The Circulatory Risk in Communities Study (CIRCS) |
| Sample source | Japan; (1) Ikawa in Akita Prefecture (northeastern rural community); (2) Noichi, Konan City in Kochi Prefecture (western rural community); and (3) Kyowa, Chikusei City in Ibaraki Prefecture (mid-eastern rural community) |
| Description | The surveyed populations comprised approximately 10,000 men and women 40 to 85 years of age who participated in cardiovascular risk surveys between 1984 and 1997 in Kyowa; between 1989 and 1995 in Ikawa and between 1989 and 1997 in Noichi. At baseline, participants underwent to a physical examination including anthropometric measurements and blood pressure and completed an extensive questionnaire about their disease history, health status, medication therapy, lifestyle, and nutritional habits. The participants were followed up to determine incident coronary heart diseases and strokes occurring by the end of 2005. For each incident coronary heart disease and stroke, 3 controls were selected by matching for sex, age (±2 years), community, year of serum storage, and fasting status at serum collection (<8 or ≥8 hours) |
| Ascertainment of fatty acid biomarker concentrations | The serum sample of 1.0 to 2.0 mL for each participant was stored at 80°C until measured. Lipids were extracted from the stored serum with chloroform and methanol and were saponified with potassium hydroxide and ethanol. Fatty acids were transesterified with BF3-methanol, and themethyl esters were analyzed in a Hitachi 263-80 gas chromatograph (Hitachi Corp) with a 3-m glass column with 3-mm internal diameter (Unisole 3000, Gas-Chro Corp). An injection temperature of 250°C, a column temperature of 220°C, and a column flow of 40 m/min of nitrogen were used. Peaks were determined by a flame ionization detector and were quantified with an electronic integrator (Hitachi Corp). Compositions of individual serum fatty acids were expressed as percentages of the total area of 13 major fatty acid peaks from 14:0 to 22:6(19). |
| Family history definition | Family history of CVD was determined from questions in paper questionnaires distributed to the participants at inclusion in the study. Individuals who reported having one or more close relatives (mother, father and siblings considered) affected by either heart attack, angina or stroke with a fatal or non-fatal outcome were considered to have a family history of CVD under definition A. The questionnaire did not specify the age of onset and whether reported diseases in close relatives needed to be clinically diagnosed. Whether siblings had to be full siblings was not specified. |
| Missing data handling | For missing covariate data, a missing indicator category was used for categorical covariates. Missing continuous covariates were handled by exclusion from the analysis. |
| CVD assessment | Defined as clinically diagnosed ischemic stroke and coronary heart disease (myocardial infarction, angina pectoris and sudden cardiac death) based on medical chart and imaging reviews. Further information was described in Yamagishi K, et al (see DOI above). |
|  | **60YO**  **doi: 10.1016/j.numecd.2006.01.002** |
| Full Name | The Stockholm Cohort of 60-year-olds (60YO) |
| Sample source | Stockholm county, Sweden; |
| Description | The 60YO is a population-based cohort including Swedish men and women, aged 60 years at the time of enrollment. From August 1997 to March 1999, every third man and woman who was born between July 1^st^ 1937 and June 30^th^ 1938 (60 years old) and living in Stockholm County, Sweden, was invited to participate in a screening for cardiovascular disease (CVD) risk factors. Among the participants invited (n=5,460), 4,232 (78% response rate), 2,039 men and 2,193 women, agreed to participate. At baseline, participants underwent to a physical examination including anthropometric measurements and blood pressure and completed an extensive questionnaire about their disease history, health status, medication therapy, lifestyle, and nutritional habits. Blood samples were also drawn after overnight fasting. Participants were followed-up for CVD and death till December 31^st^ 2017. |
| Ascertainment of fatty acid biomarker concentrations | Serum samples, collected at the time of the recruitment (1997-1999), were stored at -80°C until the analyses were performed in 2012. The percentage composition of methylated fatty acids was determined by gas-chromatography (GC) with a ﬂame ionization detector and helium as the carrier gas. To avoid contamination of the GC column, free cholesterol liberated in the reaction was removed using an aluminum oxide column. The gas-liquid chromatography (GLC) system used for the analysis consisted of a 30-m glass capillary column coated with Thermo TR-FAME (Thermo Electron Corporation,Waltham, MA, USA), and an Agilent Technologies system consisting of model GLC 6890N, an autosampler 7683 and Agilent ChemStation (Agilent Technologies Inc., Santa Clara, CA, USA). The temperature was programmed to 150–260°C. Thirteen fatty acids were identiﬁed using standards from Nu Check Prep (Elysian, MN, USA). Individual serum cholesteryl ester fatty acids were expressed as a proportion of the sum of all fatty acids measured. Fatty acid composition in one serum sample was repeatedly analyzed in duplicates in all batches for quality control and the intra- and inter-assay coefficient of variations were ≤0.24 and ≤2.49 %, respectively, for the fatty acids utilized for statistical analyses (ALA, EPA, DHA, LA, and AA). |
| Family history definition | Family history of CVD was determined from questions in paper questionnaires distributed to the participants at inclusion in the study. Individuals who reported having one or more close relatives (mother, father and siblings considered) affected by either heart attack, angina or stroke with a fatal or non-fatal outcome were considered to have a family history of CVD under definition A. Individuals who reported that their mother or father, before they turned 65 and 70, respectively, was affected by either heart attack, angina or stroke with a fatal or non-fatal outcome were considered to have a family history under definition B. Additionally classified as burdened with a family history of CVD under definition B were individuals who reported that one or more siblings had suffered from a non-fatal heart attack, angina or stroke before the age of 70 or had died from heart attack or stroke before the age of 60. The questionnaire did not specify whether reported diseases in close relatives needed to be clinically diagnosed. Whether siblings had to be full siblings were not specified. |
| Missing data handling | For missing covariate data, a missing indicator category was used for categorical covariates. Missing continuous covariates were handled by exclusion from the analysis. |
| CVD assessment | In the 60YO, CVD events were identified using the National Patient Register and the Cause of death register in Sweden. All participants were followed regarding incident of CVD and death up to 31st December 2017. During the follow-up (median 20 years), 270 men and 180 women suffered a first event of CVD. Participants that had a prior event of CVD at the baseline were excluded from the analysis (n=352). |

Table S2. Study specific definitions of family history of cardiovascular disease.

| **Study** | **Family history A (any first degree relative)** | **Family history B (age cut-off)** | **Type of CVD in the first degree relative** |
| --- | --- | --- | --- |
| **AGES-R** | Family history not specified | no | MI, stroke, hypertension |
| **KIHD** | Parents and siblings (full siblings not a requirement) | no | MI, stroke fatal and non-fatal |
| **MESA** | Parents, siblings, children | no | MI |
| **EPIC-Norfolk** | First degree relatives (full siblings not a requirement) | no | MI, stroke |
| **MCCS** | Parents and siblings (full siblings not a requirement) | no | MI, stroke |
| **EPIC-Potsdam** | Genetic parents and full siblings | cut-off <60 | MI, stroke |
| **FHS** | Parents and siblings | Cut-off: MI, CABG <55 stroke <65 | MI, CABG, stroke. Fatal CVD was used only for Fam A |
| **Hisayama** | Parents, siblings and children (full siblings not a requirement) | no | MI, stroke |
| **InCHIANTI** | Parents and siblings | no | MI, angina, or stroke |
| **WHIMS** | Parent and siblings (full siblings not a requirement) | cut off <65 | MI and stroke for fam A, MI only for Fam B |
| **CIRCS** | Parent and siblings (full siblings not a requirement) | no | MI, angina or stroke fatal or non-fatal |
| **60YO** | Parents and siblings (full siblings not a requirement) | cut off <65 male <70 female | MI, angina, stroke |
| **CHS** | Siblings only (full siblings not a requirement) | yes (no cut-off specification) | MI, stroke |
| **CRHS** | Parents and siblings (full siblings not a requirement) | cut-off <60 | MI |
| **METSIM** | Parents, siblings and children | cut-off <55 male, <65 female | CHD |

Table S3. Creation of binary variables for biomarkers of polyunsaturated fatty acids (PUFA)

| PUFA | Binary exposure variable classification |
| --- | --- |
| Low EPA and/or DHA | Any of the two fatty acids or both are ≤25^th^ percentile value.  Reference category: none of the two fatty acids are ≤25^th^ percentile value. |
| Low LA | LA is ≤25^th^ percentile value. Reference category: LA is >25^th^ percentile value. |
| Low ALA | ALA is ≤25^th^ percentile value. Reference category: ALA is >25^th^ percentile value. |

EPA: eicosapentaenoic acid; DHA: docosahexaenoic acid; LA: linoleic acid; ALA: alpha-linolenic acid

In sensitivity analyses, the ≤50^th^ percentile cut-off value was used for the corresponding variable definition.

Table S4. Distribution of n-6 PUFA linoleic acid (LA) and arachidonic acid (AA) in each of the participating studies by presence of family history (A).

|  |  |  | LA | | | | | AA | | | | |
| --- | --- | --- | --- | --- | --- | --- | --- | --- | --- | --- | --- | --- |
| Study | Family history | Lipid compartment | Mean  (SD) | Median  (IQR) | Min | Max | CV | Mean  (SD) | Median  (IQR) | Min | Max | CV |
| AGES-R |  | PL |  |  |  |  |  |  |  |  |  |  |
|  | Yes |  | 17.80(2.81) | 17.90(16.10;19.80) | 7.38 | 24.9 | 0.16 | 6.82(1.41) | 6.67(5.74;7.72) | 3.84 | 13.1 | 0.21 |
|  | No |  | 17.61(2.88) | 17.70(15.60;19.60) | 8.74 | 27.3 | 0.16 | 6.74(1.41) | 6.64(5.72;7.57) | 3.43 | 12.8 | 0.21 |
| KIHD |  | Total serum |  |  |  |  |  |  |  |  |  |  |
|  | Yes |  | 26.60(4.41) | 26.72(23.90;29.40) | 11.09 | 40.14 | 0.17 | 4.86(1.00) | 4.77(4.17;5.47) | 1.36 | 9.21 | 0.21 |
|  | No |  | 26.67(4.59) | 26.25(23.07;29.38) | 10.30 | 40.18 | 0.17 | 4.75(0.99) | 4.67(4.04;5.42) | 1.68 | 8.56 | 0.21 |
| MESA |  | PL |  |  |  |  |  |  |  |  |  |  |
|  | Yes |  | 21.10(3.20) | 21.00(19.1;23.10) | 11.40 | 35.30 | 0.15 | 12.34(2.56) | 12.14(10.62;14.06) | 6.16 | 22.17 | 0.21 |
|  | No |  | 21.80(3.39) | 21.60(19.4;24.10) | 12.10 | 36.10 | 0.16 | 11.78(2.56) | 11.65(9.90;13.53) | 5.46 | 20.51 | 0.22 |
| EPIC -Norfolk |  | PL |  |  |  |  |  |  |  |  |  |  |
|  | Yes |  | 24.29(3.45) | 24.14(21.99;26.62) | 12.19 | 38.01 | 0.14 | 9.47(1.91) | 9.36(8.1;10.62) | 4.09 | 17.88 | 0.20 |
|  | No |  | 24.32(3.41) | 24.23(21.97;26.51) | 10.80 | 40.22 | 0.14 | 9.47(1.85) | 9.39(8.17;10.63) | 3.83 | 17.3 | 0.20 |
| CHS |  | PL |  |  |  |  |  |  |  |  |  |  |
|  | Yes |  | 19.81(2.43) | 19.75(18.14;21.52) | 11.40 | 28.48 | 0.12 | 11.10(1.93) | 11.15(9.79;12.30) | 5.09 | 18.94 | 0.17 |
|  | No |  | 19.66(2.55) | 19.59(17.97;21.38) | 11.67 | 28.85 | 0.13 | 11.13(1.94) | 11.09(9.85;12.41) | 5.03 | 18.28 | 0.17 |
| CRHS |  | Adipose tissue |  |  |  |  |  |  |  |  |  |  |
|  | Yes |  | 15.52(3.75) | 15.73(12.88;17.99) | 7.60 | 30.88 | 0.24 | 0.50(0.15) | 0.49(0.39;0.59) | 0.17 | 1.03 | 0.30 |
|  | No |  | 15.35(3.85) | 15.21(12.27;17.9) | 5.40 | 28.76 | 0.25 | 0.48(0.14) | 0.47(0.38;0.57) | 0.10 | 1.06 | 0.30 |
| MCCS |  | PL |  |  |  |  |  |  |  |  |  |  |
|  | Yes |  | 20.24(2.98) | 20.13(18.24;22.14) | 10.32 | 31.16 | 0.15 | 10.39(1.82) | 10.35(9.17;11.55) | 5.01 | 18.15 | 0.17 |
|  | No |  | 20.08(2.91) | 20,00(18.23;22.03) | 9.67 | 29.26 | 0.14 | 10.39(1.73) | 10.36(9.12;11.52) | 5.79 | 17.76 | 0.17 |
| METSIM |  | PL |  |  |  |  |  |  |  |  |  |  |
|  | Yes |  | 18.87(2.72) | 18.90(16.79;20.59) | 11.68 | 28.53 | 0.14 | 8.92(1.59) | 8.77(7.69;9.95) | 5.10 | 14.43 | 0.17 |
|  | No |  | 18.49(2.63) | 18.41(16.71;20.22) | 9.61 | 26.36 | 0.14 | 9.08(1.63) | 8.92(7.96;10.07) | 4.74 | 16.8 | 0.18 |
| EPIC-Potsdam |  | RBC |  |  |  |  |  |  |  |  |  |  |
|  | Yes |  | 10.74(1.25) | 10.70(9.93;11.55) | 7.34 | 15.97 | 0.12 | 13.16(1.69) | 13.36(12.31;14.29) | 5.44 | 17.39 | 0.13 |
|  | No |  | 10.82(1.26) | 10.78(9.93;11.66) | 5.93 | 15.16 | 0.12 | 13.16(1.63) | 13.34(12.33;14.17) | 2.47 | 18.46 | 0.12 |
| FHS |  | RBC |  |  |  |  |  |  |  |  |  |  |
|  | Yes |  | 11.1(1.59) | 11.00(9.99;12.02) | 6.99 | 19.31 | 0.14 | 16.56(1.49) | 16.62(15.68;17.61) | 9.58 | 21.17 | 0.09 |
|  | No |  | 11.13(1.31) | 11.06(10.08;12.11) | 5.64 | 18.21 | 0.12 | 16.50(1.56) | 16.58(15.58;17.52) | 8.87 | 22.86 | 0.09 |
| Hisayama |  | Total serum |  |  |  |  |  |  |  |  |  |  |
|  | Yes |  | 26.77(4.53) | 26.89(23.95;29.93) | 11.89 | 41.55 | 0.17 | 4.95(0.98) | 4.91(4.25;5.61) | 1.87 | 7.97 | 0.20 |
|  | No |  | 27.14(4.52) | 27.53(24.17;30.48) | 9.34 | 38.45 | 0.17 | 5.03(1.03) | 4.99(4.34;5.68) | 1.72 | 9.51 | 0.20 |
| InCHIANTI |  | Total Plasma |  |  |  |  |  |  |  |  |  |  |
|  | Yes |  | 25.07(3.90) | 25.16(22.51;27.53) | 6.80 | 35.55 | 0.16 | 8.18(1.98) | 8.07(6.88;9.45) | 3.19 | 15.88 | 0.24 |
|  | No |  | 25.27(3.92) | 25.21(22.49;27.83) | 12.93 | 37.02 | 0.16 | 8.06(1.84) | 7.86(6.85;9.27) | 3.40 | 14.58 | 0.23 |
| WHIMS |  | RBC |  |  |  |  |  |  |  |  |  |  |
|  | Yes |  | 11.88(1.76) | 11.77(10.69;12.96) | 5.36 | 20.41 | 0.15 | 16.86(1.73) | 16.93(15.73;18.04) | 8.96 | 23.22 | 0.10 |
|  | No |  | 11.86(1.72) | 11.78(10.71;12.92) | 5.80 | 22.5 | 0.14 | 16.89(1.75) | 16.96(15.75;18.08) | 9.94 | 22.83 | 0.10 |
| CIRCS |  | Total serum |  |  |  |  |  |  |  |  |  |  |
|  | Yes |  | 25.91(4.97) | 25.90(22.77;29.56) | 8.46 | 40.51 | 0,19 | 4.58(1.15) | 4.54(3.79;5.39) | 0.22 | 8.56 | 0.25 |
|  | No |  | 26.22(5.43) | 26.10(22.75;30.14) | 10.97 | 41.02 | 0.02 | 4.42(1.14) | 4.29(3.60;5.14) | 1.92 | 8.67 | 0.26 |
| 60YO |  | CE |  |  |  |  |  |  |  |  |  |  |
|  | Yes |  | 48.40(4.10) | 48.73(46.01;51.15) | 26.41 | 62.62 | 0.08 | 6.28(1.14) | 6.21(5.50;6.94) | 3.53 | 13.28 | 0.18 |
|  | No |  | 48.55(4.28) | 48.93(45.93;53.68) | 29.30 | 62.75 | 0.09 | 6.33(1.20) | 6.23(5.52;7.02) | 3.09 | 12.70 | 0.19 |

AGES-R: Age, Gene/Environment Susceptibility-Reykjavik study; KIHD: The Kuopio Ischaemic Heart Disease Risk Factor Study; MESA: Multi-Ethnic Study of Atherosclerosis; EPIC-Norfolk: European Prospective Investigation into Cancer and Nutrition Norfolk study; CHS: Cardiovascular Health Study; CRHS: Costa Rica Heart Study; MCCS: Melbourne Collaborative Cohort Study; METSIM: The Metabolic Syndrome in Men; EPIC-Potsdam: European Prospective Investigation into Cancer and Nutrition Potsdam study; FHS: Framingham Heart Study - Offspring cohort; Hisayama: The Hisayama Study; InCHIANTI: Invecchiare in Chianti; WHIMS: Women's Health Initiative Memory Study; CIRCS: The Circulatory Risk in Communities Study; 60YO: The Stockholm Cohort of 60-year-olds.

SD: standard deviation; IQR: interquartile range; min: minimum; max: maximum; CV: coefficient of variation calculated as SD/Mean; PL: phospholipids; RBC: red blood cell; CE: Cholesteryl-esters.

Table S5. Distribution of n-3 PUFA, alfa-linolenic acid (ALA), eicosapentaenoic acid (EPA) and docosahexaenoic acid (DHA) in each of the participating studies by presence of family history (A)

|  |  |  | ALA | | | | | EPA | | | | | DHA | | | | |
| --- | --- | --- | --- | --- | --- | --- | --- | --- | --- | --- | --- | --- | --- | --- | --- | --- | --- |
| Study | Family history | Lipid compartment | Mean (SD) | Median  (IQR) | Min | Max | CV | Mean (SD) | Median  (IQR) | Min | Max | CV | Mean (SD) | Median  (IQR) | Min | Max | CV |
| AGES-R |  | PL |  |  |  |  |  |  |  |  |  |  |  |  |  |  |  |
|  | Yes |  | 0.23(0.07) | 0.22(0.18;0.27) | 0.09 | 0.75 | 0.32 | 2.75(1.53) | 2.31(1.62;3.42) | 0.55 | 10.5 | 0.56 | 6.24(1.53) | 5.97(5.11;7.14) | 3.21 | 11.6 | 0.25 |
|  | No |  | 0.23(0.08) | 0.21(0.18;0.26) | 0.09 | 0.66 | 0.34 | 2.97(1.72) | 2.47(1.69;3.77) | 0.69 | 9.68 | 0.58 | 6.38(1.53) | 6.25(5.19;7.42) | 2.5 | 11.7 | 0.24 |
| KIHD |  | Total serum |  |  |  |  |  |  |  |  |  |  |  |  |  |  |  |
|  | Yes |  | 0.72(0.23) | 0.69(0.56;0.85) | 0.24 | 1.75 | 0.32 | 1.70(0.96) | 1.49(1.10;1.99) | 0.27 | 8.67 | 0.56 | 2.47(0.75) | 2.37(1.95;2.87) | 0.94 | 6.56 | 0.30 |
|  | No |  | 0.74(0.23) | 0.72(0.58;0.87) | 0.24 | 2.06 | 0.31 | 1.62(0.82) | 1.44(1.10;1.95) | 0.23 | 8.30 | 0.51 | 2.42(0.70) | 2.33(1.95;2.78) | 0.91 | 6.58 | 0.29 |
| MESA |  | PL |  |  |  |  |  |  |  |  |  |  |  |  |  |  |  |
|  | Yes |  | 0.18(0.07) | 0.16(0.13;0.21) | 0.05 | 0.67 | 0.42 | 0.97(0.85) | 0.72(0.51;1.08) | 0.18 | 8.32 | 0.87 | 4.09(1.52) | 3.84(2.93;4.99) | 1.31 | 10.03 | 0.37 |
|  | No |  | 0.18(0.07) | 0.17(0.13;0.21) | 0.03 | 0.57 | 0.38 | 0.98(0.93) | 0.72(0.52;1.06) | 0.17 | 14.46 | 0.95 | 4.24(1.54) | 4.02(3.1;5.22) | 1.32 | 10.4 | 0.36 |
| EPIC -Norfolk |  | PL |  |  |  |  |  |  |  |  |  |  |  |  |  |  |  |
|  | Yes |  | 0.23(0.09) | 0.22(0.17;0.28) | 0.04 | 1.06 | 0.38 | 1.28(0.82) | 1.07(0.79;1.49) | 0.15 | 8.2 | 0.64 | 5.15(1.60) | 4.92(4.02;6.03) | 1.24 | 14.48 | 0.31 |
|  | No |  | 0.23(0.09) | 0.22(0.17;0.28) | 0.03 | 0.91 | 0.37 | 1.25(0.82) | 1.05(0.79;1.42) | 0.19 | 9.64 | 0.65 | 5.09(1.63) | 4.84(3.96;5.87) | 1.3 | 15.35 | 0.32 |
| CHS |  | PL |  |  |  |  |  |  |  |  |  |  |  |  |  |  |  |
|  | Yes |  | 0.15(0.05) | 0.14(0.12;0.18) | 0.05 | 0.43 | 0,33 | 0.57(0.39) | 0.5(0.38;0.66) | 0.11 | 8.52 | 0.68 | 2.97(0.93) | 2.83(2.32;3.47) | 1.08 | 6.99 | 0.33 |
|  | No |  | 0.15(0.05) | 0.14(0.11;0.18) | 0.05 | 0.47 | 0.33 | 0.61(0.37) | 0.52(0.4;0.69) | 0.11 | 4.16 | 0.61 | 3.07(0.99) | 2.91(2.36;3.58) | 1.17 | 8.17 | 0.32 |
| CRHS |  | Adipose tissue |  |  |  |  |  |  |  |  |  |  |  |  |  |  |  |
|  | Yes |  | 0.63(0.21) | 0.6(0.49;0.75) | 0.19 | 1.49 | 0.33 | 0.05(0.02) | 0.05(0.03;0.06) | 0 | 0.15 | 0.40 | 0.15(0.06) | 0.14(0.11;0.17) | 0.03 | 0.98 | 0.40 |
|  | No |  | 0.64(0.21) | 0.61(0.49;0.77) | 0.06 | 1.76 | 0.32 | 0.05(0.02) | 0.04(0.03;0.06) | 0 | 0.21 | 0.40 | 0.15(0.05) | 0.14(0.11;0.17) | 0.04 | 0.5 | 0.33 |
| MCCS |  | PL |  |  |  |  |  |  |  |  |  |  |  |  |  |  |  |
|  | Yes |  | 0.17(0.08) | 0.15(0.11;0.2) | 0.03 | 0.94 | 0.48 | 1.05(0.47) | 0.97(0.73;1.29) | 0.01 | 4.89 | 0.45 | 4.02(1.09) | 3.87(3.26;4.63) | 1.11 | 12.27 | 0.27 |
|  | No |  | 0.17(0.08) | 0.16(0.12;0.21) | 0.02 | 0.77 | 0.47 | 1.08(0.50) | 0.97(0.75;1.28) | 0.15 | 6.12 | 0.47 | 4.00(1.03) | 3.91(3.32;4.57) | 1.3 | 9.4 | 0.26 |
| METSIM |  | PL |  |  |  |  |  |  |  |  |  |  |  |  |  |  |  |
|  | Yes |  | 0.33(0.12) | 0.32(0.24;0.4) | 0.07 | 0.89 | 0.36 | 2.29(1.14) | 1.98(1.52;2.76) | 0.61 | 7.46 | 0.50 | 5.63(1.47) | 5.54(4.52;6.61) | 1.74 | 10.38 | 0.26 |
|  | No |  | 0.32(0.12) | 0.3(0.24;0.38) | 0.04 | 0.87 | 0.37 | 2.30(1.15) | 2.04(1.53;2.74) | 0.47 | 10.33 | 0.5 | 5.67(1.43) | 5.62(4.70;6.58) | 2.09 | 10.08 | 0.25 |
| EPIC-Potsdam |  | RBC |  |  |  |  |  |  |  |  |  |  |  |  |  |  |  |
|  | Yes |  | 0.16(0.11) | 0.15(0.13;0.18) | 0.06 | 2.33 | 0.67 | 0.82(0.30) | 0.78(0.60;0.97) | 0.24 | 2.51 | 0.37 | 4.82(1.08) | 4.81(4.12;5.49) | 1.47 | 8.83 | 0.22 |
|  | No |  | 0.16(0.07) | 0.15(0.13;0.18) | 0.06 | 1.09 | 0.41 | 0.81(0.30) | 0.77(0.61;0.94) | 0.08 | 2.63 | 0.37 | 4.78(1.10) | 4.78(4.09;5.48) | 0.54 | 7.87 | 0.23 |
| FHS |  | RBC |  |  |  |  |  |  |  |  |  |  |  |  |  |  |  |
|  | Yes |  | 0.18(0.09) | 0.16(0.13;0.20) | 0.04 | 1.14 | 0.49 | 0.69(0.41) | 0.59(0.45;0.79) | 0.17 | 4.92 | 0.59 | 4.7(1.34) | 4.53(3.79;5.53) | 1.43 | 10.2 | 0.29 |
|  | No |  | 0.19(0.12) | 0.16(0.13;0.21) | 0.04 | 2.04 | 0.63 | 0.73(0.49) | 0.59(0.47;0.82) | 0.2 | 6.54 | 0.67 | 4.72(1.33) | 4.59(3.81;5.47) | 1.61 | 10.21 | 0.28 |
| Hisayama |  | Total Serum |  |  |  |  |  |  |  |  |  |  |  |  |  |  |  |
|  | Yes |  | 0.69(0.20) | 0.64(0.55;0.8) | 0.24 | 2.45 | 0.30 | 2.42(1.32) | 2.14(1.46;3.08) | 0.39 | 10.22 | 54.42 | 4.84(1.30) | 4.75(3.87;5.66) | 1.24 | 11.18 | 0.27 |
|  | No |  | 0.69(0.21) | 0.65(0.54;0.8) | 0.21 | 2.16 | 0.31 | 2.27(1.25) | 2.01(1.39;2.89) | 0.26 | 12.56 | 55.24 | 4.66(1.31) | 4.51(3.77;5.42) | 1.5 | 12.73 | 0.30 |
| InCHIANTI |  | Total Plasma |  |  |  |  |  |  |  |  |  |  |  |  |  |  |  |
|  | Yes |  | 0.46(0.25) | 0.39(0.31;0.5) | 0.08 | 1.71 | 0.54 | 0.63(0.20) | 0.6(0.51;0.72) | 0.22 | 1.67 | 0.31 | 2.34(0.79) | 2.27(1.79;2.82) | 0.35 | 5.69 | 0.34 |
|  | No |  | 0.45(0.25) | 0.38(0.31;0.5) | 0.03 | 1.6 | 0.56 | 0.62(0.23) | 0.59(0.49;0.71) | 0.2 | 3.50 | 0.38 | 2.3(0.76) | 2.22(1.76;2.75) | 0.39 | 5.78 | 0.33 |
| WHIMS |  | RBC |  |  |  |  |  |  |  |  |  |  |  |  |  |  |  |
|  | Yes |  | 0.16(0.07) | 0.15(0.11;0.2) | 0.02 | 1.35 | 0.45 | 0.70(0.41) | 0.6(0.43;0.85) | 0.11 | 5.75 | 0.58 | 4.47(1.40) | 4.3(3.51;5.26) | 0.65 | 12.8 | 0.31 |
|  | No |  | 0.16(0.07) | 0.15(0.11;0.2) | 0.02 | 0.96 | 0.44 | 0.71(0.42) | 0.61(0.44;0.85) | 0.10 | 6.91 | 0.60 | 4.56(1.44) | 4.39(3.54;5.38) | 0.45 | 12.34 | 0.32 |
| CIRCS |  | Total serum |  |  |  |  |  |  |  |  |  |  |  |  |  |  |  |
|  | Yes |  | 0.97(0.49) | 0.86(0.64;1.19) | 0 | 4.67 | 0.50 | 3.56(2.04) | 3.06(2.03;4.59) | 0.48 | 13.43 | 0.57 | 4.41(1.51) | 4.33(3.32;5.25) | 0.38 | 10 | 0.34 |
|  | No |  | 0.96(0.47) | 0.86(0.63;1.16) | 0 | 3.23 | 0.49 | 3.39(1.87) | 3.02(2.06;4.33) | 0.37 | 14.69 | 0.55 | 4.41(1.48) | 4.27(3.27;5.37) | 0.54 | 8.99 | 0.33 |
| 60YO |  | CE |  |  |  |  |  |  |  |  |  |  |  |  |  |  |  |
|  | Yes |  | 0.89(0.2) | 0.87(0.75;1.00) | 0.25 | 2.00 | 0.22 | 2.11(1.01) | 1.89(1.48;2.50) | 0.51 | 13.91 | 0.48 | 0.93(0.25) | 0.90(0.76;1.06) | 0.33 | 2.5 | 0.26 |
|  | No |  | 0.88(0.21) | 0.87(0.75;1.00) | 0.24 | 2.28 | 0.24 | 2.05(0.97) | 1.85(1.40;2.41) | 0.28 | 11.6 | 0.47 | 0.90(0.25) | 0.89(0.73;1.05) | 0.25 | 2.33 | 0.28 |

AGES-R: Age, Gene/Environment Susceptibility-Reykjavik study; KIHD: The Kuopio Ischaemic Heart Disease Risk Factor Study; MESA: Multi-Ethnic Study of Atherosclerosis; EPIC-Norfolk: European Prospective Investigation into Cancer and Nutrition Norfolk study; CHS: Cardiovascular Health Study; CRHS: Costa Rica Heart Study; MCCS: Melbourne Collaborative Cohort Study; METSIM: The Metabolic Syndrome in Men; EPIC-Potsdam: European Prospective Investigation into Cancer and Nutrition Potsdam study; FHS: Framingham Heart Study - Offspring cohort; Hisayama: The Hisayama Study; InCHIANTI: Invecchiare in Chianti; WHIMS: Women's Health Initiative Memory Study; CIRCS: The Circulatory Risk in Communities Study; 60YO: The Stockholm Cohort of 60-year-olds.

SD: standard deviation; IQR: interquartile range; min: minimum; max: maximum; CV: coefficient of variation calculated as SD/Mean; PL: phospholipids; RBC: red blood cell; CE: Cholesteryl-esters.

Table S6. Distribution of the covariates race, geographical location, occupation and education for each of the participating studies by family history of CVD (A)

|  | **Race (%)** | | **Geographical location (%)** | | **Occupation (%)** | | **Education (%)** | |
| --- | --- | --- | --- | --- | --- | --- | --- | --- |
| Study | Family history | | Family history | | Family history | | Family history | |
|  | Yes | No | Yes | No | Yes | No | Yes | No |
| AGES-R | 100(White) | 100(White) | 100(Area A) | 100(Area A) | 30.6(Clerical) | 28.2(Clerical) | 23.1(<High school) | 23.5(<High school) |
|  |  |  |  |  | 64.3(Other) | 67.3(Other) | 73.0(High school graduate) | 73.7(High school graduate) |
| KIHD | 100(White) | 100(White) | 100 (Area A) | 100 (Area A) | 42.5(Clerical) | 38.9(Clerical) | 52.9( <High school) | 58.8(<High school) |
|  |  |  |  |  | 57.5(Other) | 61.1(Other) | 9.4(High school graduate) | 5.8(High school graduate) |
| MESA | 33.8(White)  13.7(Chinese American)  25.1(African American)  27.5(Hispanic American) | 23.5(White)  32.7(Chinese American)  21.4(African American)  22.4(Hispanic American) | 15.7(Wake Forest Univeristy)  17.2(Columbia)  12.5(Johns Hopkins)  17.2(Universty of Minnesota)  18.1(Northwestern University)  19.4(UCLA) | 11.8(Wake Forest Univeristy)  14.4(Columbia)  8.5(Johns Hopkins)  12.9(Universty of Minnesota)  22.0(Northwestern University)  30.4(UCLA) | 11.4(Homemaker)  44.5(Employeed Full time)  9.0(Employed Part-Time)  0.9(Employed On-Leave(Health))  0.3(Employed On-Leave(Non-Health))  1.0(Unemployed <6 months)  0.7(Unemployed >6 months)  20.3(Retired Not Working)  6.3(Retired Working)  5.3(Retired Volunteering) | 9.59(Homemaker)  49.7(Employeed Full time)  10.5(Employed Part-Time)  0.4(Employed On-Leave(Health))  0.4(Employed On-Leave(Non-Health))  0.9(Unemployed <6 months)  0.8(Unemployed >6 months)  19(Retired Not Working)  4.4(Retired Working)  4.2(Retired Volunteering) | 0.6(No Schooling)  9.4(Grades 1-8)  6.9(Grade 9-11)  19.7(Completed High School/GED)  14.8(Some College. but No Degree)  7.9(Technical School Certificate)  6.3(Associate Degree)  15.3(Bachelor's Degree)  18.8(Graduate or Professional School) | 1.0(No Schooling)  9.8(Grades 1-8)  6(Grade 9-11)  16.7(Completed High School/GED)  15.3(Some College. but No Degree)  7.5(Technical School Certificate)  5.6(Associate Degree)  19.5(Bachelor's Degree)  18.5(Graduate or Professional School) |
| EPIC-Norfolk | 100(White) | 100(White) | 100(Area A) | 100(Area A) | 6.7(Professional)  37.5(Managing)  38.2(Skilled)  14.2(Semi-skilled)  3.4(Non-skilled) | 5.9(Professional)  34.8(Managing)  41(Skilled)  14.4(Semi-skilled)  4.0(Non-skilled) | 39.3(No)  9.8(O-level)  39.5(A-level)  11.4(Degree) | 44.9(No)  8.5(O-level)  36.6(A-level)  10.0(Degree) |
| CHS | 90(White)  10(Black)  0.2(Other) | 87(White)  12(Black)  1(Other) | 28(Bowman Gray)  25(Davis)  25(Hopkins)  23(Pittsburgh) | 27(Bowman Gray)  26(Davis)  22(Hopkins)  27(Pittsburgh) | - | - | 27(<High school)  32(High school graduate)  41(College +) | 25(<High school)  27(High school graduate)  48(College +) |
| CRHS | 100(White) | 100(White) | 100 (Area A) | 100 (Area A) | 594 (434)^1^ | 534 (406) ^1^ | 4.5(No) | 6.4(No) |
|  |  |  |  |  |  |  | 51.9( <High school) | 55.0(<High school) |
|  |  |  |  |  |  |  | 26.2(High school graduate) | 24.8(High school graduate) |
|  |  |  |  |  |  |  | 17.4(>High school ) | 13.7(>High school ) |
| MCCS |  |  | 82.2(Australia/NZ/UK)  10.1(Italy)  7.7(Greece) | 69.7(Australia/NZ/UK)  15.8(Italy)  14.6(Greece) |  | - | 54.4( <High school)  22.1(High school graduate)  23.5(>College) | 56.8(<High school)  20.3(High school graduate)  22.9(>College) |
| METSIM | 100(Finnish) | 99.4(Finnish) | Kuopio area | Kuopio area | - | - | - | - |
| EPIC-Potsdam | 100(White) | 100(White) | 100(Area A) | 100(Area A) | 22.0(Clerical) | 39.9(Clerical) | 20.0(<High school. <University) | 40.3(<High school. <University) |
|  |  |  |  |  | 11.7(Other) | 26.4(Other) | 13.7(High school graduate University) | 26.0 (High school graduate University) |
| FHS | 100(White) | 100(White) | 100 (Area A) | 100 (Area A) | 45.4 (Working) | 41.7 (Working) | 2.2 (<High school) | 3.2 (<High school) |
|  |  |  |  |  | 8.7 (Homemaker) | 10.4 (Homemaker) | 25.9 (HS graduate) | 27.0 (HS graduate) |
|  |  |  |  |  | 1.0 (Unemployed)  43.2 (Retired) | 1.6 (Unemployed)  44.3 (Retired) | 21.0 (Some college;2-year degree) | 22.6 (Some college;2-year degree) |
|  |  |  |  |  |  |  | 50.3 (College graduate) | 47.0 (College graduate) |
|  |  |  |  |  |  |  |  |  |
| Hisayama | 100(Other) | 100(Other) | 100(Area A) | 100(Area A) | - | - | - | - |
| InCHIANTI | 100(White) | 100(White) | 44.6(Area A) | 53.6(Area A) | - | - | 81.8(<High school) | 76.5(<High school) |
|  |  |  |  |  |  |  | 18.2(High school graduate) | 23.5(High school graduate) |
| WHIMS | 1.8(Asian) | 2.3(Asian) | 26.1(NorthEast) | 28.2(NorthEast) | - | - | 6.8(<High school) | 7.4(<High school) |
|  | 5.2(Black) | 8.4(Black) | 21.4(South) | 20.2(South) |  |  | 93.2(High school graduate) | 92.6(High school graduate) |
|  | 2.0(Hispanic) | 2.8(Hispanic) | 24.7(Midwest) | 22.3(Midwest) |  |  |  |  |
|  | 89.5(White) | 84.3(White) | 27.7(West) | 29.2(West) |  |  |  |  |
|  | 1.5(Other) | 2.1(Other) |  |  |  |  |  |  |
| CIRCS | 100(Other) | 100(Other) | 40.5(Area A) | 34.0(Area A) | - | - | - | - |
| 60YO |  | - | - | - | - | - | 56.19(<High school)  12.8(High school)  30.7(>High school) | 56.4(<High school)  12.6(High school)  23.2(>High school) |

AGES-R: Age, Gene/Environment Susceptibility-Reykjavik study; KIHD: The Kuopio Ischaemic Heart Disease Risk Factor Study; MESA: Multi-Ethnic Study of Atherosclerosis; EPIC-Norfolk: European Prospective Investigation into Cancer and Nutrition Norfolk study; CHS: Cardiovascular Health Study; CRHS: Costa Rica Heart Study; MCCS: Melbourne Collaborative Cohort Study; METSIM: The Metabolic Syndrome in Men; EPIC-Potsdam: European Prospective Investigation into Cancer and Nutrition Potsdam study; FHS: Framingham Heart Study - Offspring cohort; Hisayama: The Hisayama Study; InCHIANTI: Invecchiare in Chianti; WHIMS: Women's Health Initiative Memory Study; CIRCS: The Circulatory Risk in Communities Study; 60YO: The Stockholm Cohort of 60-year-olds.

^1^ (mean;SD $/months)

Table S7. Distribution of covariates related to lifestyle for each of the participating studies by family history of CVD (A).

|  | **Smoking**  **(%)** | | **Physical activity**  **(mean;SD; %)** | | **Alcohol intake**  **(mean;SD)** | |
| --- | --- | --- | --- | --- | --- | --- |
| Study | Family history | | Family history | | Family history | |
|  | Yes | No | Yes | No | Yes | No |
| AGES-R | 43.3(Never) | 46.2(Never) | 18.1(Never) | 19.4(Never) | 0.17;0.36 | 0.15;0.38 |
|  | 42.9(Current) | 38.6(Current) | 19(Rarely) | 16.7(Rarely) |  |  |
|  | 11.3( Former) | 13.3(Former) | 23(Occasionally) | 23.8(Occasionally) |  |  |
|  |  |  | 22.3(Moderate) | 22(Moderate) |  |  |
|  |  |  | 17.8(High) | 18.2(High) |  |  |
| KIHD | 35.6(Never) | 33.4(Never) | 131;150(Kcal/week) | 138;185(Kcal/week) | 0.69;1.10 | 0.75;1.20 |
|  | 34.2(Current) | 34.6(Current) |  |  |  |  |
|  | 30.2(Former) | 31.9(Former) |  |  |  |  |
| MESA | 53.6(Never) | 56.7(Never) | 1096;698(METs-min/week) | 1002;684.7(METs-min/week) | 4.54;10.20 | 4.11;10.8 |
|  | 31.6(Current) | 30.58(Current) |  |  |  |  |
|  | 14.5(Former) | 12.63(Former) |  |  |  |  |
| EPIC-Norfolk | 43.6(Never) | 43.1(Never) | 34.4(Inactive) | 34.9(Inactive) | 6.91;5.01 | 4.57;6.50 |
|  | 10.1(Current) | 12.1(Current) | 28.7(Moderately inactive) | 26.9(Moderately inactive) |  |  |
|  | 46.2(Former) | 44.8(Former) | 21.0(Modierately active) | 22.1(Modierately active) |  |  |
|  |  |  | 15.8(Active) | 16.1(Active) |  |  |
| CHS | 45.6(Never) | 45.2(Never) | 1092.5; 1477.6(Kcal/week) | 1059.7; 1417.0(Kcal/week) | 1.72;4.66 | 2.20;5.15 |
|  | 45.5(Current) | 45.1(Current) |  |  |  |  |
|  | 8.9(Former) | 9.7(Former) |  |  |  |  |
| CHRS | 36.2(Never) | 35.8(Never) | 34.7;16.8 | 34.9;14.9 | 0.49;1.22 | 0.47; 1.18 |
|  | 31.9(Current) | 34.7(Current) |  |  |  |  |
|  | 31.9(Former) | 29.5(Former) |  |  |  |  |
| MCCS | 57.3(Never) | 57.1(Never) | 21.7(0) | 22.8(0) | 11.3; 18.9 | 12.3; 19.1 |
|  | 11.0(Current) | 12.8(Current) | 21.2(>0 & <4) | 20.1(>0 & <4 times/week) |  |  |
|  | 31.7 (Former) | 30.1 (Former) | 33.8(>=4 & <6) | 33.7(>=4 & <6 times/week) |  |  |
|  |  |  | 23.3(>=6) | 23.3(>=6 times/week) |  |  |
| METSIM | 43.8(Never) | 43.6(Never) | 4(A little or none) | 4.7(A little or none) | 13.7;17.3 | 15.2;17.8 |
|  | 17.9(Current)  38.3(Former) | 18.3(Current)  38.1(Former) | 25.2(Occasionally)  17.4(<=2 times a week at least 30min at a time) | 26.9(Occasionally)  18.4(<=2 times a week at least 30min at a time) |  |  |
|  |  |  | 53.3(>=3 times a week at least 30min at a time) | 50(>=3 times a week at least 30 min at a time) |  |  |
| EPIC-Potsdam | 15.9(Never) | 31.4(Never) | 2.8; 3.4(h/week) | 2.7; 3.3(h/week) | 13.2;15.7 | 14.8;21.9 |
|  | 6.8(Current) | 13.5(Current) |  |  |  |  |
|  | 11(Former) | 21.4(Former) |  |  |  |  |
| FHS | 90.5(Never)  0.2(Current)  9.2 (Former) | 90.6(Never)  0.1(Current)  9.3 (Former) | 41.9;5.97(Kcal/week) | 41.9;5.65(Kcal/week) | 23.9(None) | 25.6(None) |
|  |  |  |  |  | 48.3(Less 1 drink/day) | 49.1(Less 1 drink/day) |
|  |  |  |  |  | 21.6(1 drink/day) | 19.6(1 drink/day) |
|  |  |  |  |  | 6.0(2 or more drinks/day) | 5.3(2 or more drinks/day) |
| Hisayama | 79.4(Never or former) | 77.3(Never or former) | 35.7 (Yes) | 33.2 (Yes) | 0.9;1.6 | 1.0;1.87 |
|  | 20.6(Current) | 22.7(Current) |  |  |  |  |
| InCHIANTI | 59(Never) | 56.3(Never) | 12.90(Kcal/week) | 11.50(Kcal/week) | 1.09;1.52 | 1.20;1.55 |
|  | 16.5(Current) | 22.5(Current) |  |  |  |  |
|  | 24.5(Former) | 21.3(Former) |  |  |  |  |
| WHIMS | 54.5(Never)  7.1(Current)  37.8(Former) | 55.0(Never)  6.7(Current)  38.1(Former) | 2.52;1.13(Kcal/week) | 2.52;1.13(Kcal/week) | 45.0(non-drinker)  42.2(1-6 servings/week) | 45.0(non-drinker)  44.8(1-6 servings/week) |
| CIRCS | 50.4(Never) | 48.7(Never) | - | - | 16.6;24.1 | 13.6;22.8 |
|  | 33.9(Current) | 32.0(Current) |  |  |  |  |
|  | 15.6(Former) | 19.3(Former) |  |  |  |  |
| 60YO | 41.0(Never)  19.5(Current)  38.0(Former) | 36.0(Never)  21.0(Current)  35.0(Former) | 9.8(Inactive)  58.6(Low)  23.7(Moderate)  7(High) | 10.9(Inactive)  52.2(Low)  21.2(Moderate)  7.3(High) | 6.8(None)  62.2 (<1 drink/day)  17.6 (1-2 drinks/day)  13.2 (> 2 drinks/day | 7.8(None)  54.3 (<1 drink/day)  17.7 (1-2 drinks/day)  13.7 (> 2 drinks/day) |

AGES-R: Age, Gene/Environment Susceptibility-Reykjavik study; KIHD: The Kuopio Ischaemic Heart Disease Risk Factor Study; MESA: Multi-Ethnic Study of Atherosclerosis; EPIC-Norfolk: European Prospective Investigation into Cancer and Nutrition Norfolk study; CHS: Cardiovascular Health Study; CRHS: Costa Rica Heart Study; MCCS: Melbourne Collaborative Cohort Study; METSIM: The Metabolic Syndrome in Men; EPIC-Potsdam: European Prospective Investigation into Cancer and Nutrition Potsdam study; FHS: Framingham Heart Study - Offspring cohort; Hisayama: The Hisayama Study; InCHIANTI: Invecchiare in Chianti; WHIMS: Women's Health Initiative Memory Study; CIRCS: The Circulatory Risk in Communities Study; 60YO: The Stockholm Cohort of 60-year-olds.

MET: metabolic equivalent of task

Table S8. Distribution of covariates related to cardiometabolic risk profile and in addition aspirin treatment and cod liver/fish oil supplementation for each of the participating studies by family history of CVD (A)

|  | **Body mass-index**  **(mean;SD)** | | **Prevalent dyslipidemia**  **(%)** | | **Prevalent**  **hypertension**  **(%)** | | **Prevalent**  **diabetes**  **(%)** | | **Aspirin treatment (%)** | | **Cod liver/**  **Fish oil supplements**  **(%)** | |
| --- | --- | --- | --- | --- | --- | --- | --- | --- | --- | --- | --- | --- |
| Study | Family history | | Family history | | Family history | | Family history | | Family history | | Family history | |
|  | Yes | No | Yes | No | Yes | No | Yes | No | Yes | No | Yes | No |
|  |  |  |  |  |  |  |  |  |  |  |  |  |
| AGES-R | 26.9;4.24 | 27.3;4.44 | 16.4 | 11.9 | 82.2 | 76.4 | 25.8 | 22.5 | 40.2 | 36.9 | 62.6 | 68.9 |
| KIHD | 26.8;3.5 | 26.8;3.6 | 55.3 | 53.8 | 58.0 | 53.3 | 4.7 | 4.7 | 4.3 | 5.3 | 0 | 0 |
| MESA | 29.8;5.7 | 27.7;5.4 | 42.6 | 35.0 | 64.5 | 55.6 | 11.0 | 11.2 | - | - | - | - |
| EPIC-Norfolk | 26.5;3.8 | 26.6;3.8 | 1.7 | 0.9 | 26.0 | 21.7 | 3.6 | 3.5 | 8.9 | 7.00 | - | - |
| CHS | 26.7;4.5 | 26.8;4.7 | 21.6 | 17.8 | 56.5 | 52.4 | 16.3 | 13.6 | 29.8 | 30.3 | 2.7 | 3.7 |
| CRHS | 26.5;4.4 | 26.1;4.1 | 34.4 | 27.7 | 43.1 | 32.3 | 23.2 | 18.2 | 19.9 | 16.6 | 3.5 | 2.8 |
| MCCS | 26.9;4.5 | 26.8;4.3 | 39.5 | 35.2 | 22.9 | 17.5 | 4.4 | 6.0 | 14.9 | 11.8 | 6.6 | 5 |
| METSIM | 26.1;3.5 | 26.6;3.5 | 16.4 | 12.6 | 13.0 | 11.9 | 3.8 | 4.9 | 1.2 | 0.5 | - | - |
| EPIC-Potsdam | 26.2;4.1 | 25.8;4.1 | 9.1 | 15.1 | 11.2 | 19.0 | 0.9 | 2.1 | 3.0 | 5.0 | - | - |
| FHS | 28.3; 5.4 | 27.8; 5.4 | 38.5 | 34.8 | 43.9 | 43.4 | 12.7 | 12.9 | 39.8 | 40.0 | 14.2 | 15.7 |
| Hisayama | 23.2;3.3 | 23.0;3.4 | 10.3 | 8.4 | 28.1 | 19.7 | 20.9 | 17.6 | 2.3 | 2.5 | - | - |
| InCHIANTI | 27.2;4.0 | 26.9;4.1 | - | - | 57.3 | 49.2 | 10.6 | 8.5 | 7.4 | 6.7 | - | - |
| WHIMS | 28.2;5.4 | 28.2;5.7 | 16.3 | 13.1 | 29.0 | 23.2 | 5.23 | 5.4 | 24.1 | 20.1 | - | - |
| CIRCS | 23.2;3.2 | 23.5;3.3 | 23.9 | 26.1 | 61.2 | 53.3 | 17.0 | 15.8 | - | - | - | - |
| 60YO | 26.7;4.1 | 26.6;4.1 | 4.7 | 2.2 | 18.0 | 12.0 | 3.8 | 3.4 | 2.6 | 2.2 | 0.6 | 0.2 |

AGES-R: Age, Gene/Environment Susceptibility-Reykjavik study; KIHD: The Kuopio Ischaemic Heart Disease Risk Factor Study; MESA: Multi-Ethnic Study of Atherosclerosis; EPIC-Norfolk: European Prospective Investigation into Cancer Norfolk study; CHS: Cardiovascular Health Study; CRHS: Costa Rica Heart Study; MCCS: Melbourne Collaborative Cohort Study; METSIM: The Metabolic Syndrome in Men; EPIC-Potsdam: European Prospective Investigation into Cancer Potsdam study; FHS: Framingham Heart Study - Offspring cohort; Hisayama: The Hisayama Study; InCHIANTI: Invecchiare in Chianti; WHIMS: Women's Health Initiative Memory Study; CIRCS: The Circulatory Risk in Communities Study; 60YO: The Stockholm Cohort of 60-year-olds.

Table S9. Overview of published validation studies, based on cohorts participating in the present investigation, assessing correlation between self-reported dietary intake with biomarker levels of linoleic acid (LA), alpha-linolenic acid (ALA), eicosapentaenoic acid (EPA) and docosahexaenoic acid (DHA).

| **Cohort** | **N** | **Dietary data** | **Biomarkers compartment*** | **Adjustments** | **Correlation coefficient** | | | **Reference**  **(DOI)** |
| --- | --- | --- | --- | --- | --- | --- | --- | --- |
|  |  |  |  |  | **LA** | **ALA** | **EPA/DHA** |  |
| KIHD | 1551 | 4-day food records (% of total FA | Serum | n.a. | 0.49 | n.a. | n.a. | [10.1001/archinte.165.2.193](https://doi.org/10.1001/archinte.165.2.193) |
| MESA | 2,837 | FFQ (% of total FA) | Plasma phospholipid | age, sex, race/ethnicity, and energy intake | 0.13 | 0.05 | 0.34/0.43 | [10.1161/JAHA.113.000506](https://doi.org/10.1161/jaha.113.000506) |
| EPIC-Norfolk and  EPIC- Potsdam | 195 and 195 | FFQ; 24-HDRs (food groups, ecological level) | Plasma phospholipid | Crude | Vegetable oils  -0.13 FFQ;  0.09 24-HDRs  Nuts and seeds  0.69 FFQ;  0.53 24-HDRs  Margarines  0.43 FFQ;  0.44 24-HDRs | Vegetable oils  -0.64 FFQ;  -0.58 24-HDRs  Nuts/seed  -0.23 FFQ;  -0.51 24-HDRs  Margarines  0.78 FFQ;  0.78 24-HDRs | 0.52/0.81 FFQ;  0.65/0.80 24-HDRs | [10.3945/ajcn.2008.26834](https://doi.org/10.3945/ajcn.2008.26834) |
| MCCS | 4439 | FFQ (% of total FA) | Plasma phospholipid | Reliability coefficients of FFQ and plasma phospholipids | 0.58 (0.38, 0.73) | 0.24 (0.11, 0.35) | 0.40 (0.30, 0.49)/  0.78 (0.61, 0.88) | [10.1016/j.numecd.2006.04.005](https://doi.org/10.1016/j.numecd.2006.04.005) |
| WHIMS | 648 | FFQ (g/d) | Red blood cells | Crude | 0.09 | 0.04 | 0.38/0.43 | [10.1002/jbmr.1772](https://doi.org/10.1002/jbmr.1772) |
| 60YO | 4030 | self-reported questionnaire (food scores) | Cholesteryl-esters | Crude | 0.07 | -0.01 | 0.33/0.36 | [10.1111/jhn.12336](https://doi.org/10.1111/jhn.12336) |
| CRHS | 367 | semiquantitative FFQ | Adipose tissue | Crude | 0.36 | 0.30 | 0.15/0.18 | [10.1093/ajcn/76.4.750](https://doi.org/10.1093/ajcn/76.4.750) |
| METSIM | 10197 | FFQ (% of total FA, weighted by fatty acid) | Red blood cells | Crude | 0.16 | 0.22 | 0.41/0.34 | [10.1007/s11745-013-3832-0](https://doi.org/10.1007/s11745-013-3832-0) |

All biomarkers were expressed as % of total fatty acids (FA)

KIHD: The Kuopio Ischaemic Heart Disease Risk Factor Study; MESA: Multi-Ethnic Study of Atherosclerosis; EPIC-Norfolk: European Prospective Investigation into Cancer and Nutrition Norfolk study; EPIC-Potsdam: European Prospective Investigation into Cancer and Nutrition Potsdam study; MCCS: Melbourne Collaborative Cohort Study; WHIMS: Women's Health Initiative Memory Study; 60YO: The Stockholm Cohort of 60-year-olds. CRHS: Costa Rica Heart Study; METSIM: Metabolic Syndrome in Men.

FFQ: Food frequency questionnaire; 24-HDRs: standardized 24-h dietary recalls; n.a: not available

Figure S1. Study-specific and pooled multi-adjusted risk estimates for CVD in relation to low EPA/DHA (≤50^th^ percentile cut-off) and family history of CVD, definition A. Panel a: Presence of low EPA/DHA in absence of family history A; Panel b: Presence of family history A in absence of low EPA/DHA; Panel c: presence of low EPA/DHA and family history A.

For all panels, the reference category consists of individuals with no low EPA/DHA and no family history A. For the pooled analyses, 10,293 individuals formed the reference category of which 2,042 were CVD cases.


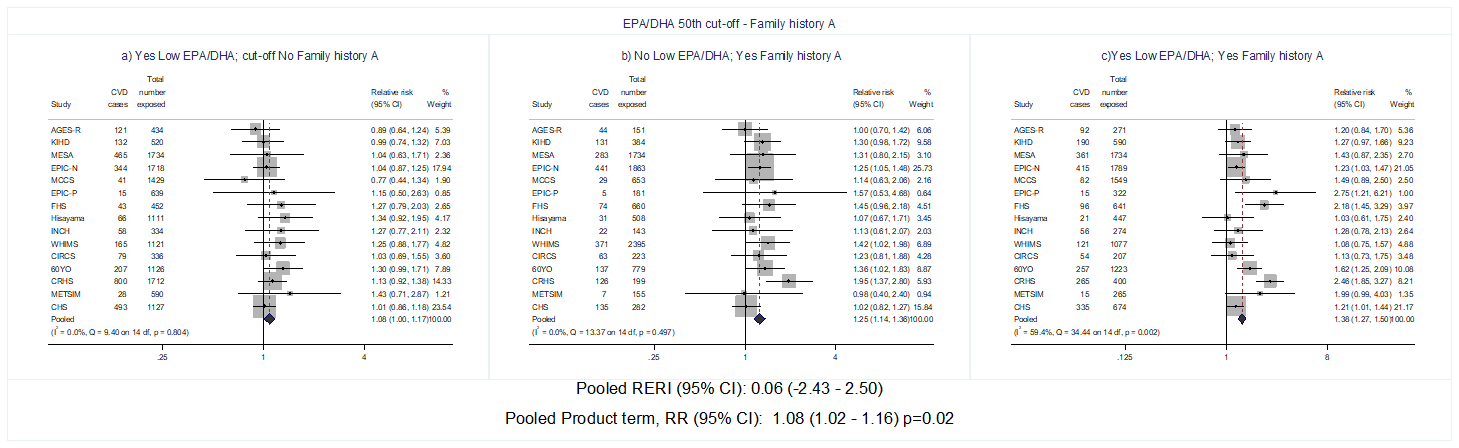


AGES-R: Age, Gene/Environment Susceptibility-Reykjavik study; KIHD: The Kuopio Ischaemic Heart Disease Risk Factor Study; MESA: Multi-Ethnic Study of Atherosclerosis; EPIC-Norfolk: European Prospective Investigation into Cancer and Nutrition Norfolk study; CHS: Cardiovascular Health Study; CRHS: Costa Rica Heart Study; MCCS: Melbourne Collaborative Cohort Study; METSIM: The Metabolic Syndrome in Men; EPIC-Potsdam: European Prospective Investigation into Cancer and Nutrition Potsdam study; FHS: Framingham Heart Study - Offspring cohort; Hisayama: The Hisayama Study; InCHIANTI: Invecchiare in Chianti; WHIMS: Women's Health Initiative Memory Study; CIRCS: The Circulatory Risk in Communities Study; 60YO: The Stockholm Cohort of 60-year-olds.

Figure S2. Study-specific and pooled multi-adjusted risk estimates for CVD in relation to low LA (≤50^th^ percentile cut-off) and family history of CVD, definition A. Panel a: Presence of low LA in absence of family history A; Panel b: Presence of family history A in absence of low LA; Panel c: Presence of low LA and family history A, respectively.

For all panels, the reference category consists of individuals with no low LA and no family history A. For the pooled analyses, 12,042 individuals formed the reference category of which 2,450 were CVD cases.


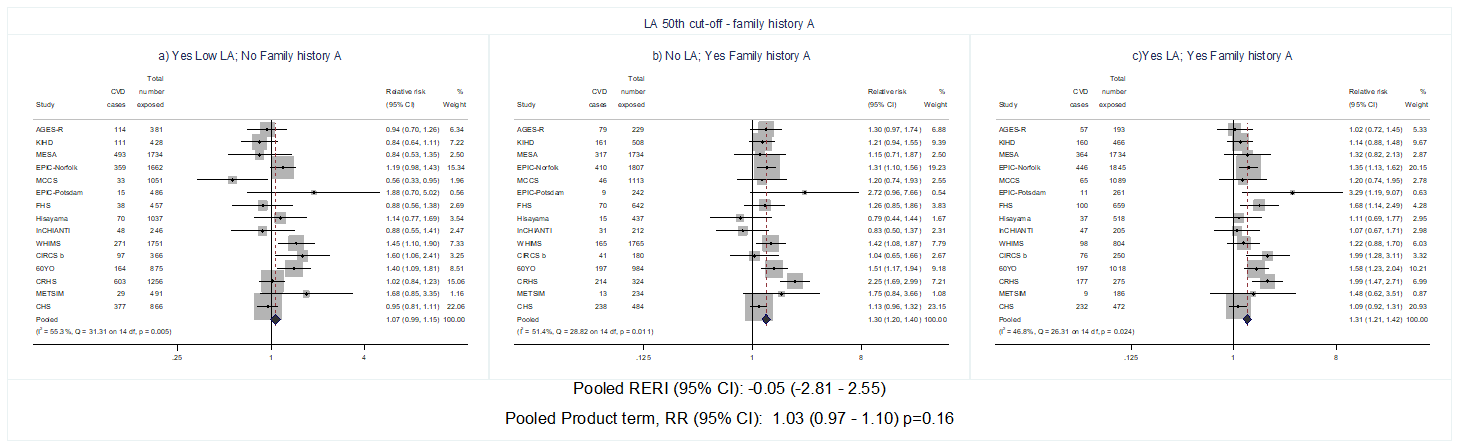


AGES-R: Age, Gene/Environment Susceptibility-Reykjavik study; KIHD: The Kuopio Ischaemic Heart Disease Risk Factor Study; MESA: Multi-Ethnic Study of Atherosclerosis; EPIC-Norfolk: European Prospective Investigation into Cancer and Nutrition Norfolk study; CHS: Cardiovascular Health Study; CRHS: Costa Rica Heart Study; MCCS: Melbourne Collaborative Cohort Study; METSIM: The Metabolic Syndrome in Men; EPIC-Potsdam: European Prospective Investigation into Cancer and Nutrition Potsdam study; FHS: Framingham Heart Study - Offspring cohort; Hisayama: The Hisayama Study; InCHIANTI: Invecchiare in Chianti; WHIMS: Women's Health Initiative Memory Study; CIRCS: The Circulatory Risk in Communities Study; 60YO: The Stockholm Cohort of 60-year-olds.

Figure S3. Study-specific and pooled multi-adjusted risk estimates for CVD in relation to low ALA (≤50^th^ percentile cut-off) and family history of CVD, definition A. Panel a: Presence of low ALA in absence of family history A; Panel b: Presence of family history A in absence of low ALA; Panel c: Presence of low ALA and family history A, respectively.

For all panels, the reference category consists of individuals with no low ALA and no family history A. For the pooled analyses, 12,063 individuals formed the reference category of which 2,425 were CVD cases.


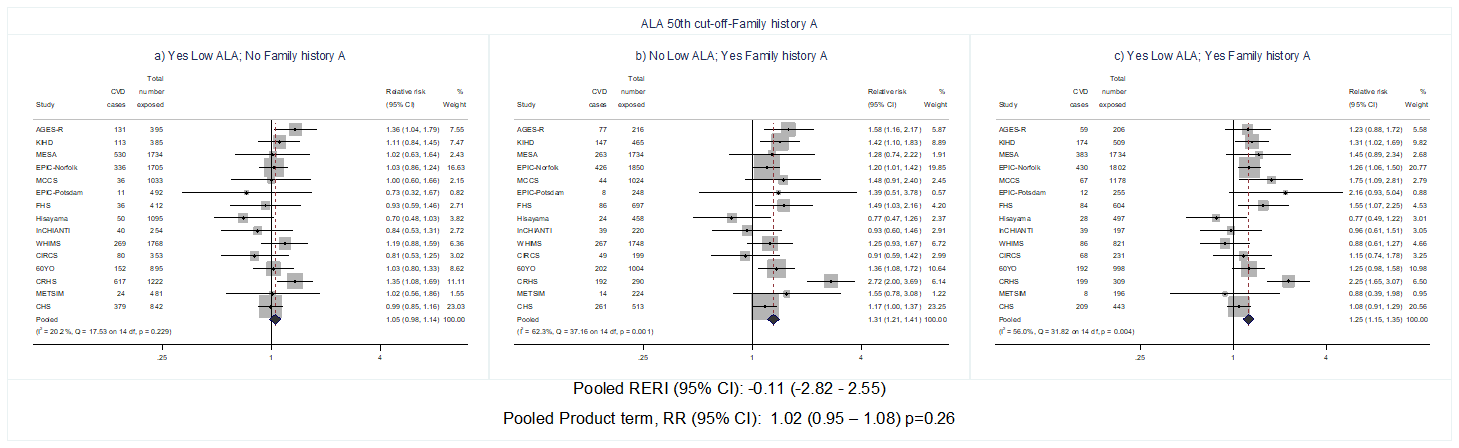


AGES-R: Age, Gene/Environment Susceptibility-Reykjavik study; KIHD: The Kuopio Ischaemic Heart Disease Risk Factor Study; MESA: Multi-Ethnic Study of Atherosclerosis; EPIC-Norfolk: European Prospective Investigation into Cancer and Nutrition Norfolk study; CHS: Cardiovascular Health Study; CRHS: Costa Rica Heart Study; MCCS: Melbourne Collaborative Cohort Study; METSIM: The Metabolic Syndrome in Men; EPIC-Potsdam: European Prospective Investigation into Cancer and Nutrition Potsdam study; FHS: Framingham Heart Study - Offspring cohort; Hisayama: The Hisayama Study; InCHIANTI: Invecchiare in Chianti; WHIMS: Women's Health Initiative Memory Study; CIRCS: The Circulatory Risk in Communities Study; 60YO: The Stockholm Cohort of 60-year-olds.

Figure S4: Study-specific and pooled multi-adjusted risk estimates for CVD in relation to low EPA/DHA (≤25^th^ percentile cut-off) and family history of CVD, definition B. Panel a: Presence of low EPA/DHA in absence of family history B; Panel b: Presence of family history B in absence of low EPA/DHA; Panel c: Presence of low EPA/DHA and family history B.

For all panels, the reference category consists of individuals with no low EPA/DHA and no family history B. For the pooled analyses, 7,807 individuals formed the reference category of which 1,696 were CVD cases. The Epic-Potsdam study is not included in panel c analysis due to few observations.

_
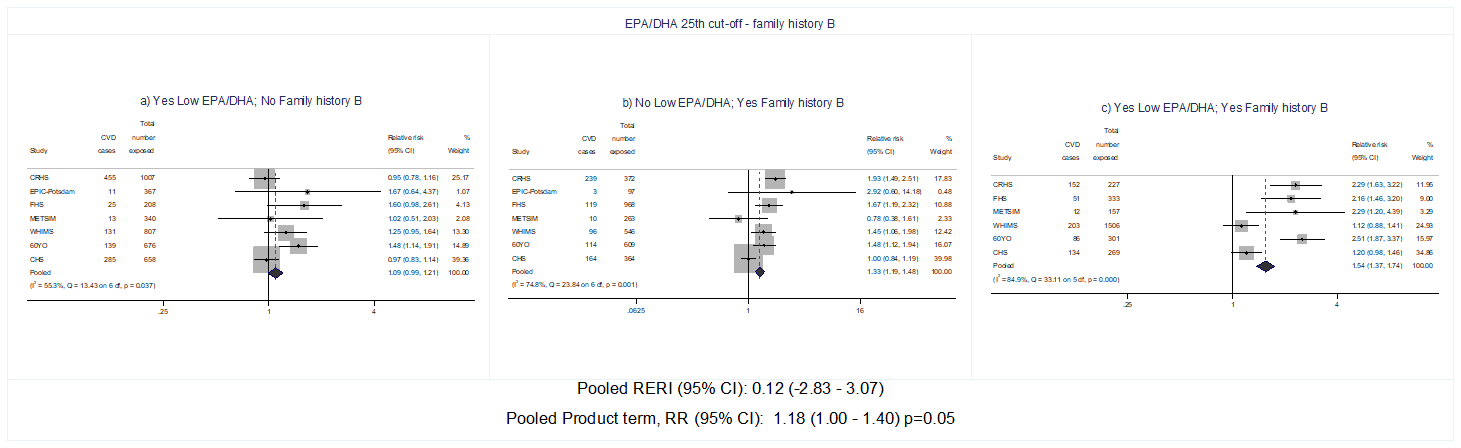
_

AGES-R: Age, Gene/Environment Susceptibility-Reykjavik study; KIHD: The Kuopio Ischaemic Heart Disease Risk Factor Study; MESA: Multi-Ethnic Study of Atherosclerosis; EPIC-Norfolk: European Prospective Investigation into Cancer and Nutrition Norfolk study; CHS: Cardiovascular Health Study; CRHS: Costa Rica Heart Study; MCCS: Melbourne Collaborative Cohort Study; METSIM: The Metabolic Syndrome in Men; EPIC-Potsdam: European Prospective Investigation into Cancer and Nutrition Potsdam study; FHS: Framingham Heart Study - Offspring cohort; Hisayama: The Hisayama Study; InCHIANTI: Invecchiare in Chianti; WHIMS: Women's Health Initiative Memory Study; CIRCS: The Circulatory Risk in Communities Study; 60YO: The Stockholm Cohort of 60-year-olds.

Figure S5. Study-specific and pooled multi-adjusted risk estimates for CVD in relation to low LA (≤25^th^ percentile cut-off), and family history of CVD, definition A. Panel a: Presence of low LA in absence of family history B; Panel b: Presence of family history B in absence of low LA; Panel c: Presence of low LA and family history B.

For all panels, the reference category consists of individuals with no low LA and no family history B. For the pooled analyses, 9,401 individuals formed the reference category of which 2,056 were CVD cases.


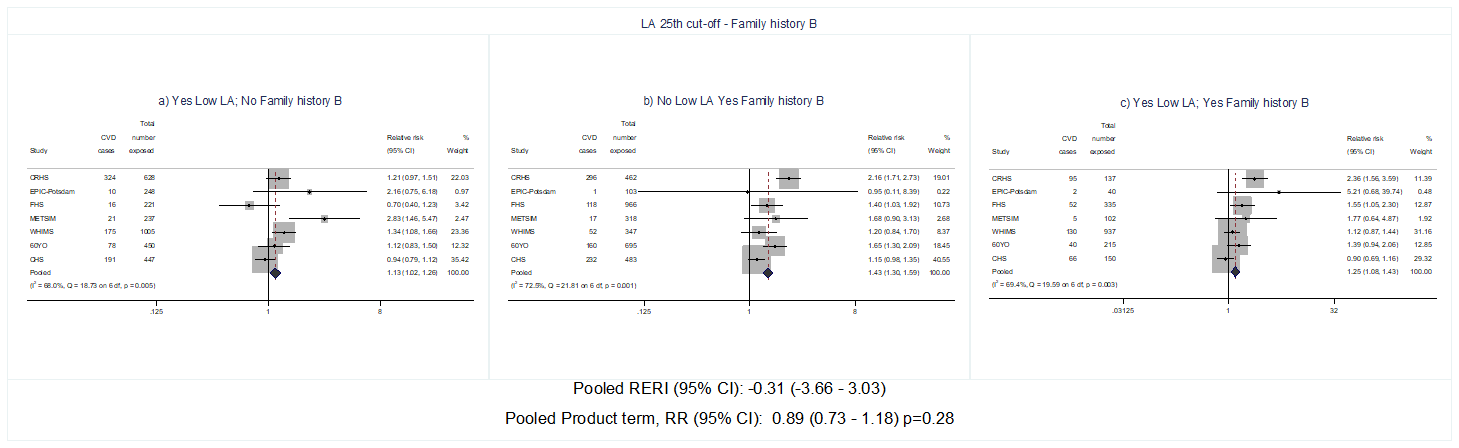


AGES-R: Age, Gene/Environment Susceptibility-Reykjavik study; KIHD: The Kuopio Ischaemic Heart Disease Risk Factor Study; MESA: Multi-Ethnic Study of Atherosclerosis; EPIC-Norfolk: European Prospective Investigation into Cancer and Nutrition Norfolk study; CHS: Cardiovascular Health Study; CRHS: Costa Rica Heart Study; MCCS: Melbourne Collaborative Cohort Study; METSIM: The Metabolic Syndrome in Men; EPIC-Potsdam: European Prospective Investigation into Cancer and Nutrition Potsdam study; FHS: Framingham Heart Study - Offspring cohort; Hisayama: The Hisayama Study; InCHIANTI: Invecchiare in Chianti; WHIMS: Women's Health Initiative Memory Study; CIRCS: The Circulatory Risk in Communities Study; 60YO: The Stockholm Cohort of 60-year-olds.

Figure S6. Study-specific and pooled multi-adjusted risk estimates for CVD in relation to low ALA (≤25th percentile cut-off) and family history of CVD, definition B. Panel a: Presence of low ALA in absence of family history B; Panel b: Presence of family history B in absence of low ALA; Panel c: Presence of low ALA and family history B.

For all panels, the reference category consists of individuals with no low ALA and no family history B. For the pooled analyses, 9,428 individuals formed the reference category of which 2,056 were CVD cases. The Epic-Potsdam study is not included in the panel c analysis due to few observations.

**
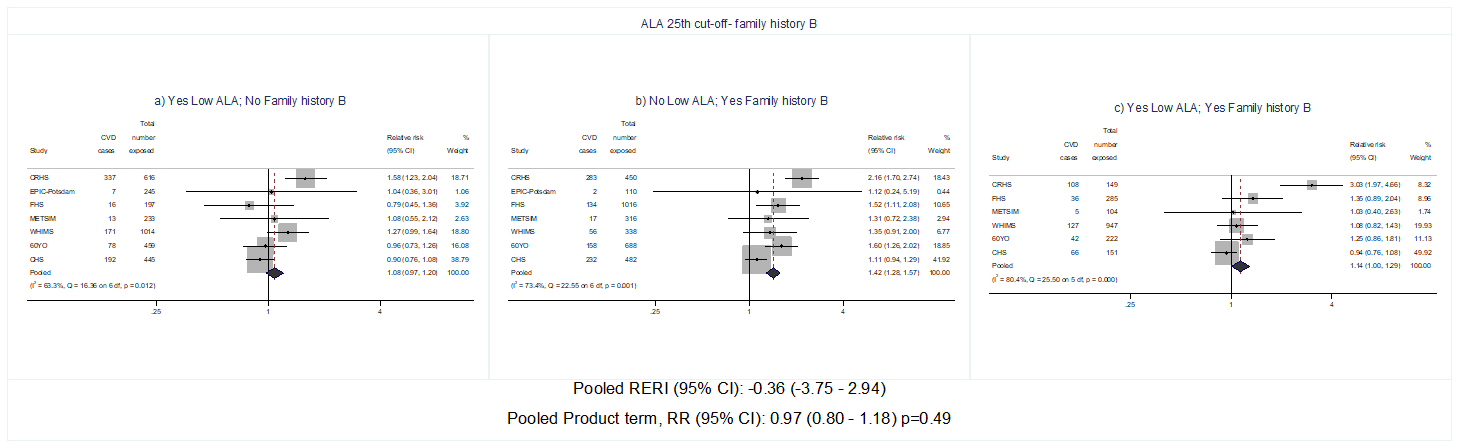
**

AGES-R: Age, Gene/Environment Susceptibility-Reykjavik study; KIHD: The Kuopio Ischaemic Heart Disease Risk Factor Study; MESA: Multi-Ethnic Study of Atherosclerosis; EPIC-Norfolk: European Prospective Investigation into Cancer and Nutrition Norfolk study; CHS: Cardiovascular Health Study; CRHS: Costa Rica Heart Study; MCCS: Melbourne Collaborative Cohort Study; METSIM: The Metabolic Syndrome in Men; EPIC-Potsdam: European Prospective Investigation into Cancer and Nutrition Potsdam study; FHS: Framingham Heart Study - Offspring cohort; Hisayama: The Hisayama Study; InCHIANTI: Invecchiare in Chianti; WHIMS: Women's Health Initiative Memory Study; CIRCS: The Circulatory Risk in Communities Study; 60YO: The Stockholm Cohort of 60-year-olds.

Figure S7. Study-specific and pooled multi-adjusted risk estimates for CVD in relation to low EPA/DHA (≤50^th^ percentile cut-off) and family history of CVD, definition B. Panel a: Presence of low PUFA_fish,_ in absence of family history B; Panel b: Presence of family history B in absence of low EPA/DHA; Panel c: Presence of low EPA/DHA_,_ and family history A.

For all panels, the reference category consists of individuals with no low EPA/DHA and no family history B. For the pooled analyses, 4,280 individuals formed the reference category of which 906 were CVD cases.


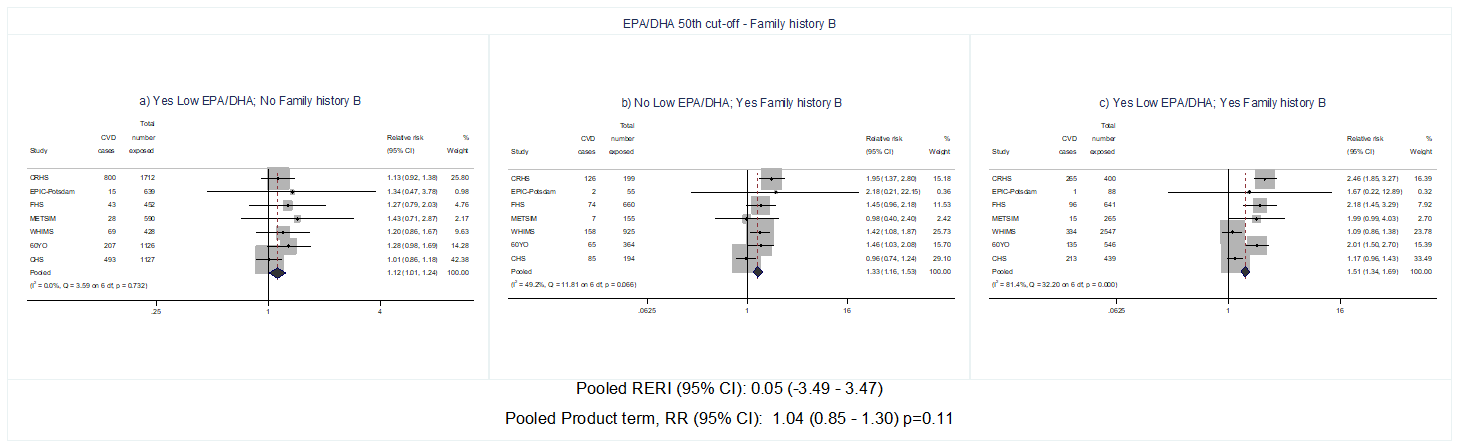


AGES-R: Age, Gene/Environment Susceptibility-Reykjavik study; KIHD: The Kuopio Ischaemic Heart Disease Risk Factor Study; MESA: Multi-Ethnic Study of Atherosclerosis; EPIC-Norfolk: European Prospective Investigation into Cancer and Nutrition Norfolk study; CHS: Cardiovascular Health Study; CRHS: Costa Rica Heart Study; MCCS: Melbourne Collaborative Cohort Study; METSIM: The Metabolic Syndrome in Men; EPIC-Potsdam: European Prospective Investigation into Cancer and Nutrition Potsdam study; FHS: Framingham Heart Study - Offspring cohort; Hisayama: The Hisayama Study; InCHIANTI: Invecchiare in Chianti; WHIMS: Women's Health Initiative Memory Study; CIRCS: The Circulatory Risk in Communities Study; 60YO: The Stockholm Cohort of 60-year-olds.

Figure S8. Study-specific and pooled multi-adjusted risk estimates for CVD in relation to low LA (≤50^th^ percentile cut-off) and family history of CVD, definition B. Panel a: Presence of low LA in absence of family history B; Panel b: Presence of family history B in absence of low LA; Panel c: Presence of low LA and family history B (panel c).

For all panels, the reference category consists of individuals with no low LA and no family history B. For the pooled analyses, 6,259 individuals formed the reference category of which 1,345 were CVD cases. The Epic-Potsdam study is not included in panel b analysis due to few observations.


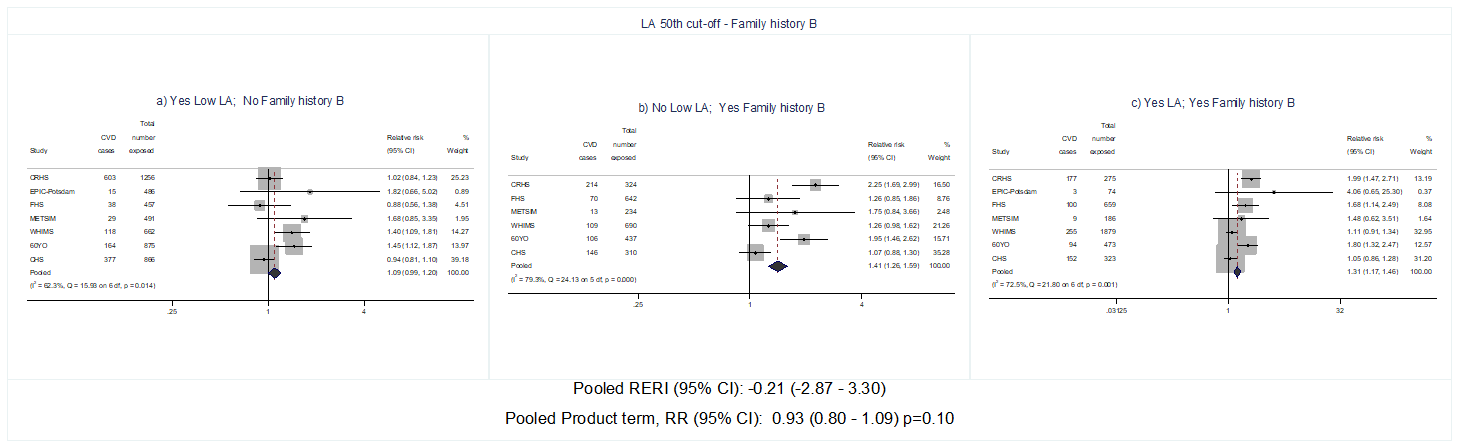


AGES-R: Age, Gene/Environment Susceptibility-Reykjavik study; KIHD: The Kuopio Ischaemic Heart Disease Risk Factor Study; MESA: Multi-Ethnic Study of Atherosclerosis; EPIC-Norfolk: European Prospective Investigation into Cancer and Nutrition Norfolk study; CHS: Cardiovascular Health Study; CRHS: Costa Rica Heart Study; MCCS: Melbourne Collaborative Cohort Study; METSIM: The Metabolic Syndrome in Men; EPIC-Potsdam: European Prospective Investigation into Cancer and Nutrition Potsdam study; FHS: Framingham Heart Study - Offspring cohort; Hisayama: The Hisayama Study; InCHIANTI: Invecchiare in Chianti; WHIMS: Women's Health Initiative Memory Study; CIRCS: The Circulatory Risk in Communities Study; 60YO: The Stockholm Cohort of 60-year-olds.

Figure S9. Study-specific and pooled multi-adjusted risk estimates for CVD in relation to low ALA (≤50th percentile cut-off) and family history of CVD, definition B. Panel a: Presence of low ALA in absence of family history B, Panel b: Presence of family history B in absence of low ALA; Panel c: Presence of low ALA and family history B, respectively.

For all panels, the reference category consists of individuals with no low ALA and no family history B. For the pooled analyses, 6,332 individuals formed the reference category of which 1,365 were CVD cases. The Epic-Potsdam study is not included in panel b analysis due to few observations.


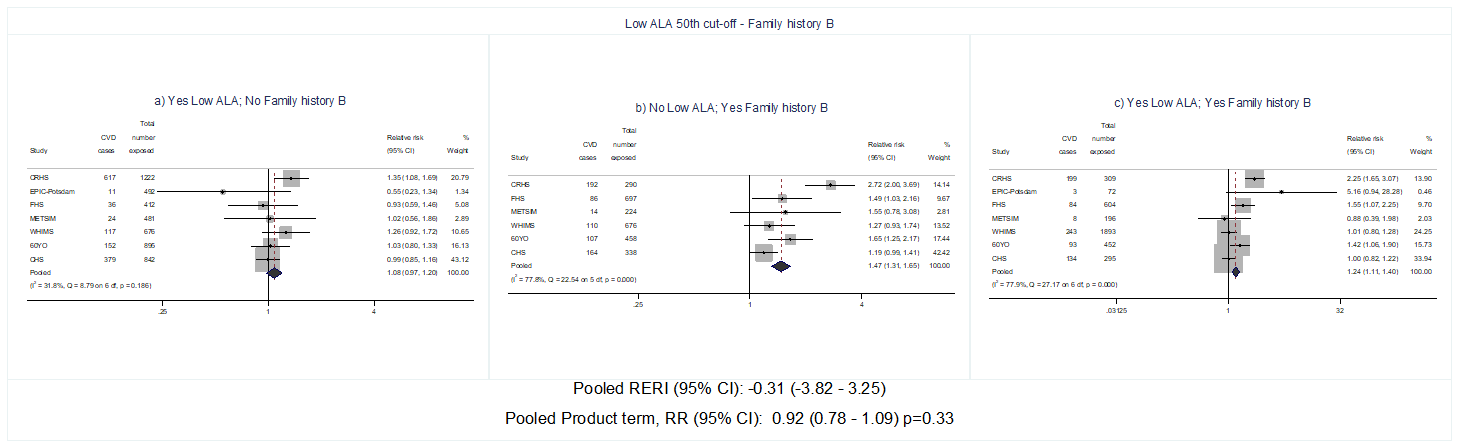


AGES-R: Age, Gene/Environment Susceptibility-Reykjavik study; KIHD: The Kuopio Ischaemic Heart Disease Risk Factor Study; MESA: Multi-Ethnic Study of Atherosclerosis; EPIC-Norfolk: European Prospective Investigation into Cancer and Nutrition Norfolk study; CHS: Cardiovascular Health Study; CRHS: Costa Rica Heart Study; MCCS: Melbourne Collaborative Cohort Study; METSIM: The Metabolic Syndrome in Men; EPIC-Potsdam: European Prospective Investigation into Cancer and Nutrition Potsdam study; FHS: Framingham Heart Study - Offspring cohort; Hisayama: The Hisayama Study; InCHIANTI: Invecchiare in Chianti; WHIMS: Women's Health Initiative Memory Study; CIRCS: The Circulatory Risk in Communities Study; 60YO: The Stockholm Cohort of 60-year-olds.

**Study specific funding information**

AGES-R: The Age, Gene/Environment Susceptibility-Reykjavik study is supported by The Office of Dietary Supplements, NIH contract N01-AG012100, the NIA Intramural Research Program, Hjartavernd (the Icelandic Heart Association), the Althingi (the Icelandic Parliament), Canadian Cancer Society (grant #704735) and the Michael Smith Foundation for Health Research (#17644)

KIHD: The Kuopio Ischaemic Heart Disease Risk Factor study is supported mainly by the funding from the Academy of Finland to Jukka T. Salonen.

MESA: The Multi-Ethnic Study of Atherosclerosis project is conducted and supported by the National Heart, Lung, and Blood Institute (NHLBI) in collaboration with MESA investigators. Support for MESA is provided by contracts 75N92020D00001, HHSN268201500003I, N01-HC-95159, 75N92020D00005, N01-HC-95160, 75N92020D00002, N01-HC-95161, 75N92020D00003, N01-HC-95162, 75N92020D00006, N01-HC-95163, 75N92020D00004, N01-HC-95164, 75N92020D00007, N01-HC-95165, N01-HC-95166, N01-HC-95167, N01-HC-95168, N01-HC-95169, UL1-TR-000040, UL1-TR-001079, UL1-TR-001420. Also supported in part by the National Center for Advancing Translational Sciences, CTSI grant UL1TR001881, and the National Institute of Diabetes and Digestive and Kidney Disease Diabetes Research Center (DRC) grant DK063491 to the Southern California Diabetes Endocrinology Research Center.

EPIC-Norfolk: The European Prospective Investigation into Cancer and Nutrition Norfolk study has received funding from the Medical Research Council (MR/N003284/1 and MC-UU_12015/1) and Cancer Research UK (C864/A14136). NJW, NGF, and FI is supported by the Medical Research Council Epidemiology Unit core funding [MC_UU_12015/1 and MC_UU_12015/5]. NJW and NGF acknowledge support from the National Institute for Health Research Cambridge Biomedical Research Centre [IS-BRC-1215-20014] and NJW is an NIHR Senior Investigator.

CHS: The Cardiovascular Health Study is supported by NHLBI contracts HHSN268201200036C, HHSN268200800007C, HHSN268201800001C, N01HC55222, N01HC85079, N01HC85080, N01HC85081, N01HC85082, N01HC85083, N01HC85086, 75N92021D00006; and NHLBI grants U01HL080295, R01HL087652, R01HL105756, R01HL103612, R01HL120393, and U01HL130114, R01HL085710 with additional contribution from the National Institute of Neurological Disorders and Stroke (NINDS). Additional support is provided through R01AG023629 from the National Institute on Aging (NIA). A full list of principal CHS investigators and institutions can be found at CHS-NHLBI.org/. The content is solely the responsibility of the authors and does not necessarily represent the official views of the National Institutes of Health.

CRHS: The Costa Rica Heart Study is supported by the National Institutes of Health [HL49086, HL60692], USA.

MCCS: The Melbourne Collaborative Cohort Study cohort recruitment is funded by VicHealth and Cancer Council Victoria. The MCCS is further augmented by Australian National Health and Medical Research Council grants 209057, 396414 and 1074383 and by infrastructure provided by Cancer Council Victoria. Vital status was ascertained through the Victorian Cancer Registry and the Australian Institute of Health and Welfare, including the National Death Index.

METSIM: The Metabolic Syndrome in Men Study is funded by the grants from The European Union, the Academy of Finland, and the Juselius Foundation.

EPIC-Potsdam: The European Prospective Investigation into Cancer and Nutrition Potsdam study is supported by the German Federal Ministry of Science (01 EA 9401) and the European Union (SOC 95201408 05F02) for the recruitment phase of the EPIC-Potsdam Study. The follow-up of the EPIC-Potsdam Study was supported by the German Cancer Aid (70-2488-Ha I) and the European Community (SOC 98200769 05F02). This work was furthermore supported by a grant from the German Ministry of Education and Research (BMBF) and the State of Brandenburg (DZD grant 82DZD00302) and by a grant from the German Research Foundation (DFG, SCHU 1516/5-1).

FHS: The Framingham Heart Study is conducted and supported by the National Heart, Lung and Blood Institute (NHLBI; R01 HL089590) and in collaboration with Boston University (Contract No. N01-HC-25195).

Hisayama: The Hisayama study was supported in part by Grants-in-Aid for Scientific Research A (JP16H02692), B (JP17H04126, JP18H02737, and JP19H03863), and C (JP18K07565, JP18K09412, JP19K07890, JP20K10503, and JP20K11020), Grants-in-Aid for Early-Career Scientists (JP18K17925 and JP19K19474), and a Grant-in-Aid for Research Activity Start-up (JP19K23971) from the Ministry of Education, Culture, Sports, Science and Technology of Japan; by Health and Labour Sciences Research Grants of the Ministry of Health, Labour and Welfare of Japan (20FA1002); and by grants from the Japan Agency for Medical Research and Development (JP20dk0207025, JP20km0405202, and JP20fk0108075).

InCHIANTI: The InCHIANTI study baseline (1998-2000) was supported as a "targeted project" (ICS110.1/RF97.71) by the Italian Ministry of Health and in part by the U.S. National Institute on Aging (Contracts: 263 MD 9164 and 263 MD 821336); the InCHIANTI Follow-up 1 (2001-2003) was funded by the U.S. National Institute on Aging (Contracts: N.1-AG-1-1 and N.1-AG-1-2111); the InCHIANTI Follow-ups 2 and 3 studies (2004-2010) were financed by the U.S. National Institute on Aging ( Contract: N01-AG-5-0002);supported in part by the Intramural Research Program of the National Institute on Aging, National Institutes of Health, Baltimore, Maryland

WHIMS: The Women's Health Initiative Memory Study program is funded by the NHLBI/NIH through contracts HHSN268201600018C, HHSN268201600001C, HHSN268201600002C, HHSN268201600003C, and HHSN268201600004C. RBC fatty acids were measured under contract BAA19.

CIRCS: The Circulatory Risk in Communities Study is supported by Osaka Prefecture, the Ministry of Education, Culture, Sports, Science and Technology, the Ministry of Health, LaboUr, and Welfare

60Y0: The Stockholm Cohort of 60-year-olds is supported by Stockholm County Council, Swedish Heart and Lung-Foundation, Swedish Research Council, The Swedish Research Council for Longitudinal Research, ALF, The Cardiovascular Program in Stockholm, The Strategic Research in Epidemiology at Karolinska Institutet.

**Study specific acknowledgements:**

AGES-R: The authors thank Pho Diep for technical assistance with fatty acid analyses.

MESA: The authors thank the other investigators, the staff, and the participants of the MESA study for their valuable contributions. A full list of participating MESA investigators and institutions can be found at http://www.mesa-nhlbi.org

CHS: The authors express their gratitude to the CHS participants.

CRHS: The authors are indebted to the Costa Rica Heart Study fieldworkers for their effort and dedication and to the participants of the study.

MCCS: The MCCS was made possible by the contribution of many people, including the original investigators, the teams that recruited the participants and continue working on follow-up, and the many thousands of Melbourne residents who continue to participate in the study.

METSIM: The authors express their gratitude to the METSIM participants.

EPIC-Potsdam: The authors thank the Human Study Centre (HSC) of the German Institute of Human Nutrition Potsdam-Rehbrücke, namely the trustee and the data hub for the processing, and the participants for the provision of the data, the biobank for the processing of the biological samples and the head of the HSC, Manuela Bergmann, for the contribution to the study design and leading the underlying processes of data generation. We thank Eugéne Jansen and Hans Cremers for the excellent performance of the FA analysis.

FHS: The authors express their gratitude to the FHS participants.

Hisayama: The authors thank the staff of the Division of Health and Welfare of Hisayama for their cooperation in this study.

InCHIANTI: The authors express their gratitude to the InChianti participants.

WHIMS: The authors thank the WHI investigators and staff for their dedication, and the study participants for making the program possible. A listing of WHI investigators can be found at <https://www.whi.org/doc/WHI-Investigator-Long-List.pdf>

60YO: The authors thank all study participants. The authors also thank Mai-Lis Hellénius for her contribution to establishing the Stockholm Cohort of 60-year-olds and Tommy Cederholm for his contribution to making the fatty acid analyzes possible.
